# Deammonification Potential of Pig Slurries and Vapor Condensates from Sewage Sludge Drying—Substrate Quality and Inhibition

**DOI:** 10.3390/bioengineering10070826

**Published:** 2023-07-11

**Authors:** Johannes Reiter, Maike Beier

**Affiliations:** Institute of Sanitary Engineering and Waste Management (ISAH), Faculty of Civil Engineering and Geodetic Science, Leibniz University Hannover, Welfengarten 1, 30167 Hanover, Germany; beier@isah.uni-hannover.de

**Keywords:** nitrogen removal, pig slurry, vapor condensate, characterization, inhibition, sewage sludge drying

## Abstract

Deammonification is a well-established process for sludge liquor treatment and promising for wastewaters with high nitrogen loads because of its low energy demand compared to nitrification/denitrification. Two wastewaters with high NH_4_-N concentrations and a rising significance in Germany—pig slurry (12 samples) and condensates from sewage sludge drying (6 samples)—were studied for their deammonification potential. Furthermore, a comprehensive quality assessment is presented. Both wastewaters show a wide range in terms of COD_t_, COD_s_, TN and NH_4_-N, whereby condensates show a greater variability with no direct relation to dryer type or temperature. In the slurries, COD_t_ shows a relative standard deviation of 106% (mean 21.1 g/L) and NH_4_-N of 33% (mean 2.29 g/L), while in condensates it reaches 148% for COD_t_ (mean 2.0 g/L) and 122% for NH_4_-N (mean 0.7 g/L). No inhibition of ammonium-oxidizing-bacteria was detected in the slurries, while two out of five condensates showed an inhibition of >40%, one of >10% and two showed no inhibition at all. Since the inhibition could be avoided by mixing, deammonification can be recommended for condensate treatment. For slurry treatment, the importance of employing some form of solid-liquid-separation as a pretreatment was noted due to the associated COD.

## 1. Introduction

In the year 2017, two amendments affecting the agricultural sector and the wastewater sector came into force in Germany. The ordinance redefining best practice in the application of fertilizer and the new sewage sludge ordinance increase the significance for slurry/manure management and treatment as well as the treatment of vapor condensates from sewage sludge drying. In Lower Saxony, Germany, the need for the treatment of pig slurry is most prevalent. Even though pig slurry and vapor condensates differ in origin and general composition, both are potentially highly loaded with ammonium-nitrogen (NH_4_-N), a main concern in wastewater treatment. Pig slurry can contain up to 5 g/L NH_4_-N [1,2,3], while condensates from sewage sludge drying can comprise more than 3 g/L NH_4_-N [4]. These high nitrogen concentrations seem well fitted for a recovery of nitrogen and its subsequent use as fertilizer or base chemical, advancing nutrient recycling and circular economy. But due to its high energy demand a nitrogen recovery, such as stripping and rectifying, is not always economically feasible. In that case, a nitrogen-removal treatment is preferable. The most commonly used process for nitrogen removal in municipal wastewater treatment is nitrification/denitrification. The high nitrogen concentrations of pig slurries and vapor condensates can, however, pose a problem for this process, demanding a different one [5]. Additionally, the ratio of biodegradable Chemical Oxygen Demand (COD_b_) to nitrogen (N) of 5.8 necessary for an optimal nitrification/denitrification as recommended by Bonassa, Bolsan et al. [6] is seldom reached in vapor condensates and pig slurries, as this study shows. This translates to a poorly functioning denitrification, which is dependent on sufficient carbon for nitrate reduction. As Ahmed, Rind and Rani [7] point out in their review, the addition of an external carbon source is often key to a well-functioning denitrification process and can drastically increase its efficiency. One of the main drivers in operating cost for a nitrification/denitrification process, however, is the cost for such an external carbon source [8]. Vineyard, Hicks et al. [9] report the annual operating costs for a nitrification/denitrification process to be 2.6 times higher compared to a carbon-independent process such as anaerobic ammonium oxidation. To reduce operating costs, efforts are being made to assess the potential of using carbon-loaded wastes as an external carbon source. Mahmoud, Hamza and Elbeshbishy [10] even considered possible part streams from wastewater treatment plants (WWTP) such as fermentation filtrates from the fermentation of primary sludge or thickened waste-activated sludge. But even with a reduction in procurement costs due to the use of waste carbon, the efficiency of the process stays reliant on external factors. Another key factor in nitrification/denitrification operation costs is the high oxygen demand due to the necessity to oxidize all present ammonium to nitrate [9].

The deammonification process, on the other hand, is well suited for wastewaters highly loaded with N and low in degradable Chemical Oxygen Demand (COD) [6], and well established for the treatment of municipal part streams such as sludge liquor from dewatering [11].

The process of deammonification consists of two steps. In the first step, partial nitritation, ammonium-oxidizing bacteria (AOB) convert approximately half of the ammonium present in the wastewater to nitrite (NO_2_). In the second step, anaerobic ammonium oxidation (anammox), NH_4_-N and NO_2_-N are directly converted into diatomic nitrogen (N_2_) and water by planctomycetoa [12]. As all the bacteria involved in the deammonification process engage in autotrophic respiration, there is no need for an available carbon source present in the wastewater. The lesser need for ammonium oxidization also results in energy savings due to a lessened oxygen demand. However, the possibility of nitrous oxide emissions needs to be checked, especially in two-stage deammonification plants [13].

Currently, studies on nitrogen elimination in pig slurries or manures focus on anaerobically treating (co-)digested pig wastewaters. For this stream, different treatment methods are being investigated. This includes nitritation/denitritation with a lower need for degradable COD and energy than nitrification/denitrification [14] and deammonification, consisting of partial nitritation/anammox [15,16,17,18,19,20,21,22]. Because of the competition for nitrogen between comparatively fast-growing denitrifying bacteria and anammox bacteria, an environment with high concentrations of Total Organic Carbon (TOC) can limit the anammox process [16,21]. To address this issue, a deammonification coupled with some form of COD elimination was investigated more recently, with promising results [23,24,25,26]. Also, inhibitions by digestate of anaerobically treated pig manure on the deammonification process have been reported [22]. In circumstances where a treatment of pig slurry is necessary for nitrogen removal but pre-treatment via anaerobic digestion is not feasible—mainly in small scale piggery operations—a deammonification of the untreated or separated pig slurry may be of use. No studies could be found addressing the deammonification of undigested pig slurries.

Vapor condensates from sewage sludge drying—mainly deriving from middle- or high-temperature drying—are usually treated with the main stream at a wastewater treatment plant. Even if full drying is implemented the condensates make up only about 10% of the process water originating from sludge treatment (dewatering and drying) [27]. While the hydraulic charge into the main stream is not of great concern, the concentrated nutrients can pose problems for the effectiveness of the pre-existing treatment methods and reduce the elimination efficiency of the overall plant. The increased quantity of condensates that arises where sewage sludge from different WWTP is dried collectively may overstrain the treatment capacity of the WWTP receiving the condensates [28]. With an increasing number of mono-incineration plants and the reception of sewage sludge from different WWTP for drying at one facility the significance of the treatment of the condensates before recharge into the WWTP rises. For a separate treatment of condensates Döllerer and Wilderer [29] propose a treatment via nitrification/denitrification in a Sequencing Batch Biofilm Reactor (SBBR) to reach the influent quality of the WWTP. The use of SBBR minimizes the negative impact of high NH_4_-N concentrations, low COD concentrations and varying influent qualities. Still, COD in the form of methanol needed to be supplied in testing [29]. These concerns can also be met with the deammonification adding the benefits of a lower energy demand for aeration and eliminating the need for degradable COD in the process. Separately treating vapor condensates may result in inhibitions of a biological treatment, e.g., through heavy metals such as zinc, nickel or copper [30]. Beier, Mahnig et al. [27] reported inhibitory effects on AOB in three out of five condensate samples, with up to a 45% reduction in AOB activity.

A problem in planning any treatment for pig slurry or vapor condensates is the variability in their quality and composition. There seems to be a lack of comprehensive overviews on both pig slurry and vapor condensate quality and their systematic research on broader range of influencing factors in the international literature. The composition of pig slurry, the animal’s diet, the stabling (slatted floor, straw), and possible anaerobic conversion in storage [31] as well as the amount of water used for cleaning the pig pens and any unused food can have a major influence on slurry composition [32]. Still seldom are these factors reported in studies dealing with the treatment of pig slurries, making it difficult to assess the mentioned compositions. In vapor condensates, the type of dryer and its operation (degree of drying, temperature) are not the only influences on the condensate composition. Also, the degree of anaerobic stabilization and the degree of dewatering of the sludge before drying influences NH_4_-N and total COD (COD_t_) concentrations. The COD_t_ is also highly influenced by any de-dusting stages [28]. Additionally, the type of condenser used influences the composition of the condensate with a diluting effect in spray condensers using treated condensate or process water for condensation [27,33]. As with pig slurry, often the peripheral aggregates and operating setting are not mentioned satisfactorily for a good classification of reported values.

This study therefore aims for a characterization and possible categorization of pig slurries and condensates from sewage sludge drying by analyzing 12 pig slurries and 6 condensates with a focus on COD, NH_4_-N and total alkalinity (TA) for assessing their deammonifcation potential as well as presenting an overview over characteristics reported in the literature. Also, possible inhibition effects—other than that of pH and free ammonia (FA)—to the first step of deammonification (nitritation—AOB activity) induced by the use of pig slurry or condensates are investigated. With this, a better understanding of expectable concentrations and possible ranges in piggery and sewage sludge drying operations is achieved. Additionally, conclusions for further process development can be drawn.

## 2. Materials and Methods

### 2.1. Pig Slurry Collection

For this study, a total of 12 pig slurries from 12 different farms (one sample from each farm) in Lower Saxony, Germany, were collected. The samples differ in terms of animal, feed, stabling and point of collection. In total, 10 samples originated from the finishing of pigs, 1 sample from the rearing of piglets and 1 sample from sow keeping. While all farms used slatted floors in their stables, one farm also employed straw following the guidelines for an animal welfare initiative (“Initiative Tierwohl”). The feed was mainly standard feed except for one farm where N-P-reduced feed was used. Half the samples could be collected freshly in the central channel, while the other 6 samples were collected from the storage tank where the slurries are stored for several weeks. Table 1 gives an overview of the collected slurry samples and their origin.

Additionally, S-1 was monitored over a period of 9 months (November 2021–August 2022) with a sampling every 3–14 days (the exception is a 21-day break in February) to evaluate any seasonal variations. 

### 2.2. Collection of Vapor Condensates

For the characterization of vapor condensates, 6 samples were collected from different municipal WWTPs across Germany that employ a sewage sludge drying facility. Furthermore, two data sets were collected. A waste incineration plant, which also dries and incinerates sewage sludge from several different WWTPs in their vicinity, supplied the results of a test run of drying vapor condensation. The other data set includes the data of three years (2018, 2021, 2022) of condensate monitoring of a large WWTP in northern Germany. Table 2 gives an overview of the condensate samples used in this study and their origin regarding drying method, drying temperature and dry matter content after drying. Except for one, all facilities dried exclusively anaerobically digested sewage sludge with some WWTP receiving additional co-substrates. Only the facility, where the data for C-7 was collected, supplied their dryer with about 7% of aerobically stabilized sludge.

### 2.3. Physiochemical Characterization

All samples, both slurries and condensates, were stored at 4 °C after their collection and analyzed in the following 1–3 days. Several physicochemical parameters were considered to assess the quality of the samples. These include the total solids (TS) and volatile solids (VS) for slurries, totals suspended solids (TSS) and volatile suspended solids (VSS) for condensates, loss on ignition (LOI), pH, total Chemical Oxygen Demand (COD_t_), soluble Chemical Oxygen Demand (COD_s_), total nitrogen (TN), ammonium nitrogen (NH_4_-N), nitrite nitrogen (NO_2_-N), nitrate nitrogen (NO_3_-N), total phosphorous (TP), orthophosphate phosphorous (PO_4_-P) and total alkalinity (TA). While the comparatively low amount of particles in the condensate samples allowed for a TSS measurement, the high amount of solids in the pig slurries hindered the use of this method. Therefore, TS was considered the best parameter to assess the amount of solids in the slurries. TS, VS, TSS, VSS and LOI were executed as described in the standard DIN 38409-1 [34] with the exception that for the TSS measurement a filter with a pore size of 0.45 µm was used. Electrical Conductivity (EC) and pH were measured using a handheld combined pH/EC meter. For the other parameters, appropriate cuvette tests were used in order to allow for a photometric measurement. In preparation for COD_t_, TN and TP analysis the samples were homogenized using a laboratory disperser. For NH_4_-N, NO_2_-N, NO_3_-N, PO_4_-P and TA the samples were filtered using a paper filter (<4 µm). To measure COD_s_, an additional filtration using a membrane filter (0.45 µm) was employed. For easier reading, a list of all parameters used in this study (for measurement or discussion) with their associated abbreviation and unit is presented in Table A1 in Appendix A.

### 2.4. Inhibition on Nitritation

**Figure 1:**
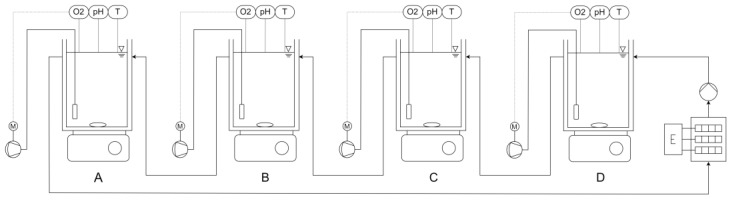
Schematic illustration of the experimental set up for inhibition assessment with the four reactors A, B, C and D, controllable air pumps (marked M), electric heating (marked E) and the associated measurements of DO (O2), pH and temperature (T).

To assess possible inhibitory effects of the slurries and condensates on a biological treatment—in this case the first step of the deammonification, the nitritation—batch tests based on the experimental set up used by Beier, Mahnig et al. [27] were conducted, comparing the maximum activity (measured as Oxygen Uptake Rate—OUR) reached with different substrate compositions. For each slurry/condensate sample, 4 reactors with a volume of 2 L were filled with 1.8 L of activated sludge originating from a large-scale single-stage deammonification plant in Germany and pre-aerated overnight to eliminate any remaining ammonium or degradable COD and to determine any endogenic respiration. Preliminary, the sludge was diluted to 1.2–1.3 g/L VSS in order not to overwork the aeration system. Each reactor was equipped with an aeration stone, a pH electrode and an optical dissolved oxygen (DO) sensor, heated to 26 °C using a circulation thermostat and stirred continuously. Figure 1 shows the experimental set up used in this study.

The aeration system was set to start the aeration when the DO concentration in the reactor undershot 2 mgO2/L and stop aerating when 4 mgO2/L where reached. In the following unaerated phase, the time it took for the DO concentration to fall from 3.8 mgO2/L to 2.2 mgO2/L was registered to calculate the OUR of that particular aeration cycle as the delta in DO concentration divided by the delta in time [mgO2/L/h]. With this intermittent aeration, the development of the OUR indicating AOB activity could be registered over the course of the experiment. Each reactor received a different composition of substrate consisting of the slurry/condensate sample in different dilutions with tab water (TW) maintaining the same volume and maintaining the same NH_4_-N-load by adding ammonium chloride (NH_4_Cl). (A) received 100% of slurry/condensate, (B) received 50% slurry/condensate and 50% tap water supplemented with NH_4_Cl to assess any effects of dilution on a possible inhibitory substance, (C) received 100% of slurry/condensate but also 86 µmol/L allylthiourea (ATU) to inhibit the nitrification and register any heterotrophic respiration and (D) received 100% of sample volume in NH_4_Cl-topped tab water as a reference. The sample volume was calculated to ensure a sludge loading (SL) of 0.06 gN/gVSS and differed in each experiment. After pre-aeration, the stirring was stopped and following a settling period supernatant was drawn to make room for the slurry/condensate sample. After adding the substrates to the reactors, the pH was adjusted to 7.5–8 and manually held in this range over the time of the experiment. The combination of set sludge loading and biomass resulted in an initial NH_4_-N concentration of approx. 75 mg/L. To ensure for an optimal nitritation process, acid capacity in form of sodium bicarbonate (NaHCO_3_) was added to each reactor. Table 3 shows the settings for an inhibition batch test with 4 reactors.

The experiments were run until a continuous decrease in OUR indicated substrate depletion. To be able to only compare the autotrophic respiration rates responsible for nitrification, the heterotrophic OUR registered in reactor (C) was subtracted fully and partially (50%) from the registered OUR in (A) and (B), respectively. In each reactor, the VSS concentration was determined following DIN 38409-1 (DIN) to calculate the Specific Oxygen Uptake Rate (SOUR) for each reactor [mgO2/gVSS/h]. To assess any inhibition the maximum SOUR reached in reactor (A) and (B) were compared to the maximum SOUR in the reference (D). The maximum SOUR being the mean SOUR registered for a prolonged period at the highest level. Any negative deviation of the maximum SOUR of greater than 5%, compared to the reference, was considered an inhibition.

#### Experimental Setting for C-1

Because of the low NH_4_-N concertation in C-1, it was not possible to ensure a sludge loading of 0.06 gN/gVSS for the inhibition test without diluting the sludge to an unreasonable degree. Therefore, the setting was done differently than described above. In accordance with the other experiments, reactor A received an undiluted sample of C-1 resulting in an unusually low sludge loading. Instead of supplying reactor B with a 1:2 diluted sample of C-1, reactor B received an undiluted sample of C-1 topped with NH_4_Cl to match the sludge loading of 0.06 gN/gVSS. The reference (D) was also set to a sludge loading of 0.06 gN/gVSS to be comparable with the other batch tests.

## 3. Results

### 3.1. Characterization and of Examination of Pig Slurry

#### 3.1.1. Composition of Pig Slurries

In the considered pig slurries TS, COD_t_, COD_s_, TN and NH_4_-N vary widely. TS ranges from 5.4 g/kg to 125.1 g/kg, COD_t_ from 3.13 to 89.50 g/L, COD_s_ from 0.79 to 19.40 g/L, TN from 0.72 to 8.84 g/L and NH_4_-N from 0.65 to 3.26 g/L. The COD_s_ makes up between only 1% and 88% of the COD_t_, also showing a great variability. High values for TS not only suggest a high COD_t_ but also a tendency of high TN and TP which can be observed in the considered data. LOI lies between 51.2% and 81.9% originating from different amounts of inorganic material in the slurries, underlining the influence of stabling, washing and storage on slurry composition stated by Møller, Sommer et al. [31] and Choi, Kim et al. [32]. While NO_3_-N was detected with maximum 35.8 mg/L, NO_2_-N was only detected in one slurry (0.5 mg/L). The pH is the most stable parameter with a range from 7.16 to 8.07.

Even though a high COD_t_ is present in all slurries, it does not translate directly into a degradable fraction, for some of it is linked to larger particles. To estimate the biodegradability, COD_s_ is used instead. The ratio of COD_s_:NH4-N ranges from 0.1 to 6.6. Using COD_s_ as a substitute for COD_b_, only 1 out of 12 samples reaches the recommended ratio of 5.8 for nitrification/denitrification, encouraging the use of a less COD-dependent treatment. For the deammonification to function optimally the anammox needs a ratio of NH_4_-N to NO_2_-N of 1.32 [35]. This means approximately 56% of the NH_4_-N in the slurry needs to be oxidized to NO_2_-N. As the nitritation process releases protons (H+) and no protons are bound in further steps as is the case in denitrification, sufficient total alkalinity (TA) is crucial to a stable deammonification. For each milligram of NH_4_-N converted, 0.14 mmol of TA are used up [36]. So, to ensure a conversion of 56% of NH_4_-N, a minimum TA:NH_4_-N ratio of 0.0784 mmol/mg in the wastewater is needed. In the considered slurries, TA:NH_4_-N ranges from 0.07 mmol/mg to 0.12 mmol/mg allowing for a theoretical oxidation of at least 56% of the NH_4_-N present in 11 out of 12 samples. 

All data on the slurries including TS, LOI, pH, COD_t_, COD_s_, TN, NH_4_-N, NO_2_-N, NO_3_-N, TP, PO_4_-P and TA can be found in Table A2 in Appendix A. Further comparison of the measured COD_t_, COD_s_ and NH_4_-N with the literature and slurry monitoring is presented under Section 3.1.3.

#### 3.1.2. Pig Slurry Monitoring

**Figure A1:**
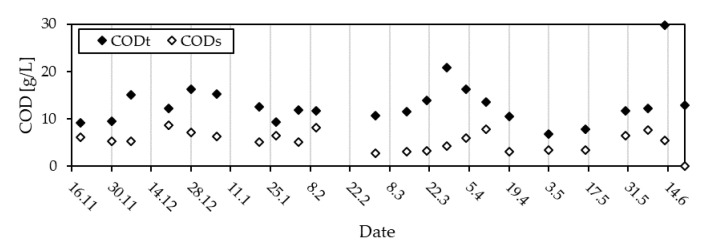
Measured COD_t_ and COD_s_ in pig slurry over the 9-month monitoring period.

**Figure A2:**
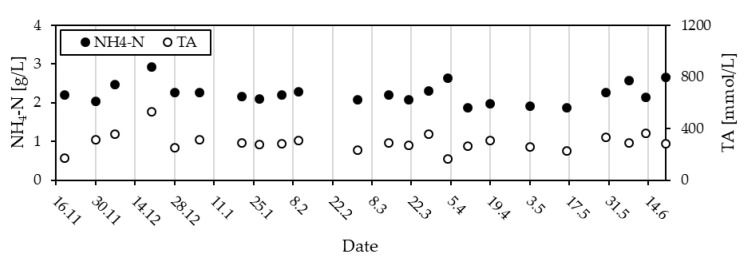
Measured NH_4_-N and TA in pig slurry over the 9-month monitoring period.

**Figure A3:**
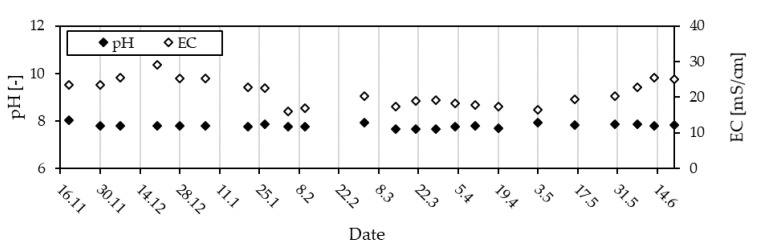
Measured pH and EC in pig slurry over the 9-month monitoring period.

The pig slurry shows a rather broad variation in almost every parameter over the course of the 9-month monitoring period. The pH is an expectation, with a relative standard deviation of only 1.3%. Table 4 gives a comprehensive overview of the parameters of the slurry monitoring with the minimum and maximum recorded in the 9 months as well as the arithmetic mean, standard deviation (SD), relative standard deviation (%RSD) and number of samples. While COD_t_ follows the pattern described by the TS, COD_s_ varies independently. On average, the COD_s_ made up 43.6% of the COD_t_, with the LOI averaging at 50.5%. Except for NO_2_-N and NO_3_-N, all measurements fit well with the results of the slurry characterization. The maximum for NO_2_-N lies at 2.79 mg/L and for NO_3_-N at 67.00 mg/L. The COD_s_:NH_4_-N ratio ranges from 1.3 to 4.1 with an average of 2.4, not reaching the recommended 5.8 for nitrification/denitrification. TA:NH_4_-N ratio varies between 0.06 to 0.18 mmol/mg with an average of 0.13 mmol/mg allowing for a theoretical oxidation of over 56% of the present NH_4_-N in 28 of 30 samples. Over the course of the monitoring period, EC and TA seem to be following the pattern described by NH_4_-N. Even though a broad variation in almost all parameters is present, no clear seasonal pattern as described by Choi, Kim et al. [32] could be observed. Figure A1, Figure A2 and Figure A3 in Appendix A show the distribution of the COD_t_, COD_s_, NH_4_-N, TA, pH and EC over the 9-month monitoring period. All measured values can be found in the Supplementary Materials in Table S1.

#### 3.1.3. Slurry Quality—An Overview of COD_t_, COD_s_ and NH_4_-N

**Figure 2:**
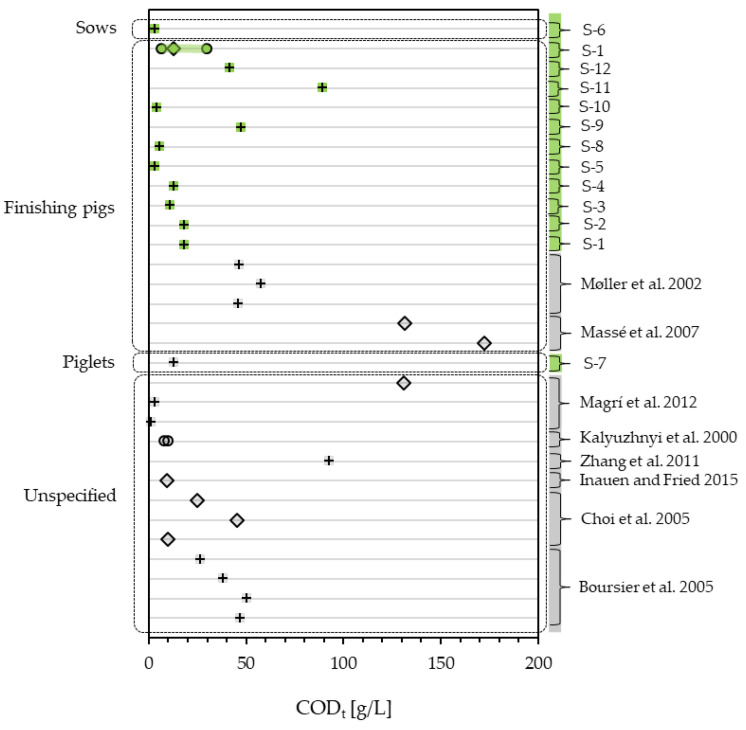
COD_t_ of pig slurry measured in this study (green) compared to the literature values (grey) [2,31,32,38,40,41,42,43], differentiating between one-time samples (+), mean values (◊) and ranges (o).

The results from the slurry characterization and monitoring obtained in this study show a broad range in different parameters. Assessing the deammonification potential COD, NH_4_-N and TA is most important. For TA, only 5 values could be obtained in the literature and linked to NH_4_-N [2,37,38], with only one sample not reaching the TA:NH_4_-N ratio of 0.0784 mmol/mg [2]. COD_t_ values in this study range from 3.13 g/L (S-5) up to 89.50 g/L (S-11), both being one-time samples. The monitoring data show that even a continuous piggery operation produces a wide range in COD_t_ from 6.775 to 29.750 mg/L with an average of 13.144 g/L. With this, the measured COD_t_ fits the literature well. Figure 2 shows the measured COD_t_ in this study set in context with the literature values. In the literature, COD_t_ ranges between 1.453 g/L [38] and 172.39 g/L [39]. Still, 14 of 18 values in the literature lie under 60 g/L [32,40,41,42], which is where the monitoring data and 11 of the 12 studied samples fit in. Sample S-11 with 89.50 g/L, as well as four values in the literature (92.8 g/L [2], 130.8 g/L [38], 131.3 and 172.89 mg/L [39]), seem to be exceptionally highly loaded with COD_t_. 

**Figure 3:**
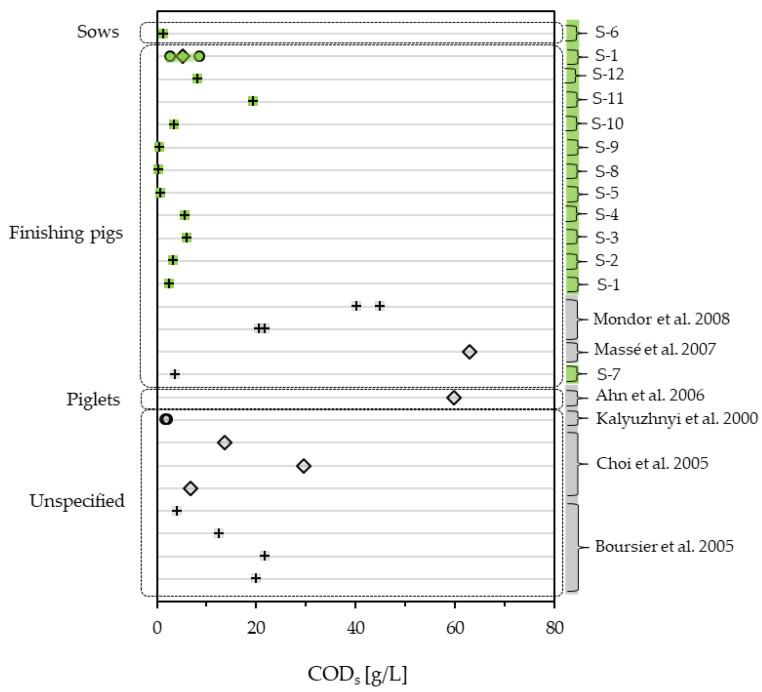
COD_s_ of pig slurry measured in this study (green) compared to literature values (grey) [32,40,42,43,45,46], differentiating between one-time samples (+), mean values (◊) and ranges (o).

Using solid–liquid separation, a good reduction in COD_t_ is often achievable [44]. This potential is apparent in the literature and the examined samples, with COD_s_ making up as little as 13.7% (S-1) or 15.9% [40] of COD_t_. An extreme finding is sample S-9, with only 1.0% of the COD_t_ being COD_s_. Considering COD_s_, values up to 59.7 g/L and 62.98 g/L are reported in literature [39,45]. The majority of values reported in the literature (10 of 14) lie under 30 g/L [32,40,42,46], as do all the examined samples. Compared to the literature, the examined samples still score low, with 11 of 12 samples being under 10 g/L and the highest scoring at 19.4 g/L COD_s_. Figure 3 shows the measured COD_s_ values in comparison to the values found in the literature.

**Figure 4:**
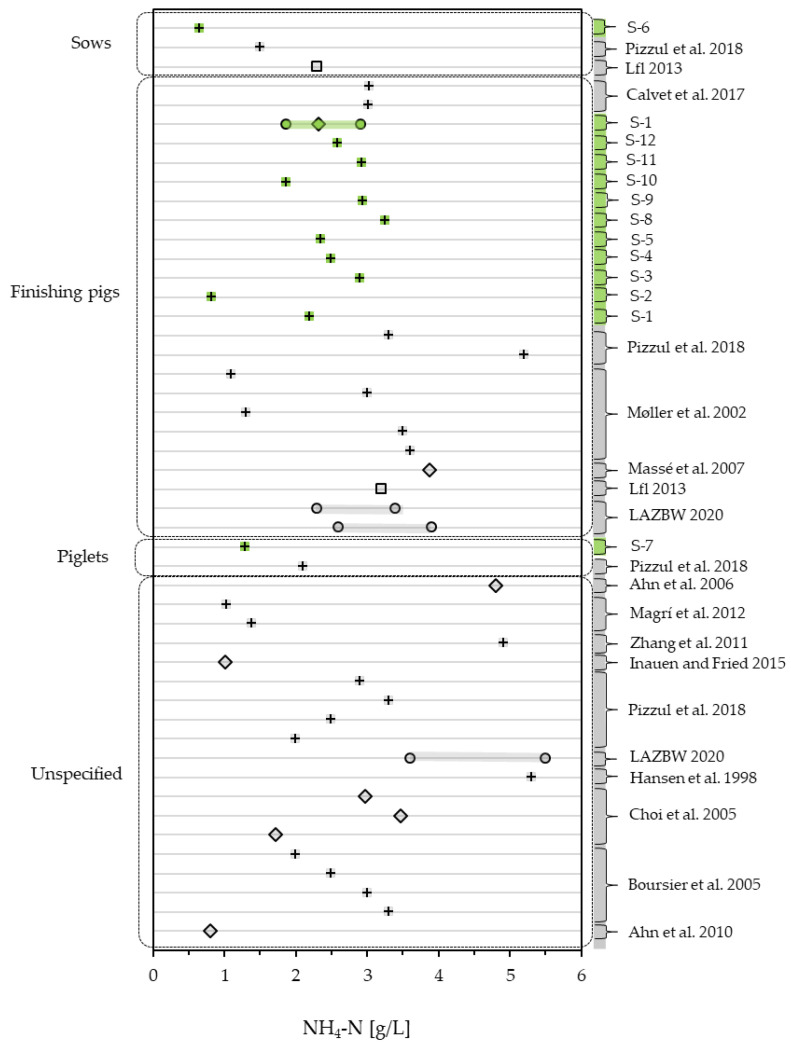
NH_4_-N of pig slurry measured in this study (green) compared to literature values (grey) [1,2,3,31,32,37,38,40,41,43,45,47,48,49], differentiating between one-time samples (+), mean values (◊), ranges (o) and medians (□).

NH_4_-N does not vary as vastly as COD_t_ and COD_s_ but still shows a broad variation. The examined samples range from 0.65 g/L (S-6) to 3.26 g/L (S-8), while the range found in the literature goes from 0.802 g/L [37] up to 5.5 g/L [47]. Of the 48 considered samples (12 samples + monitoring mean + 35 literature values), 8 lie below 1.5 g/L [31,37,38,41], while the majority (35 of 48) score between 1.5 and 4 g/L [3,31,32,40,43,47,48,49]. This includes the range observed in the slurry monitoring (1.870–2.916 g/L), as well as the ranges reported by the agricultural center of Baden–Wuerttemberg (LAZBW) [47] for finishing pigs (2.6–3.9 g/L and 2.3–3.4 g/L). Only five values found in the literature exceed 4 g/L [1,2,3,45,47] with one being the high end of the range reported by LAZBW [47] for pig rearing. Figure 4 compares the NH_4_-N content of the examined slurries to the values found in the literature.

Often the animal type, feeding and stabling conditions are not directly or consistently mentioned in literature. Due to insufficient data, no quantitative connection between the influencing factors and COD_t_, COD_s_ or NH_4_-N in pig slurry can be derived.

#### 3.1.4. Inhibition Testing with Pig Slurries

**Figure A4:**
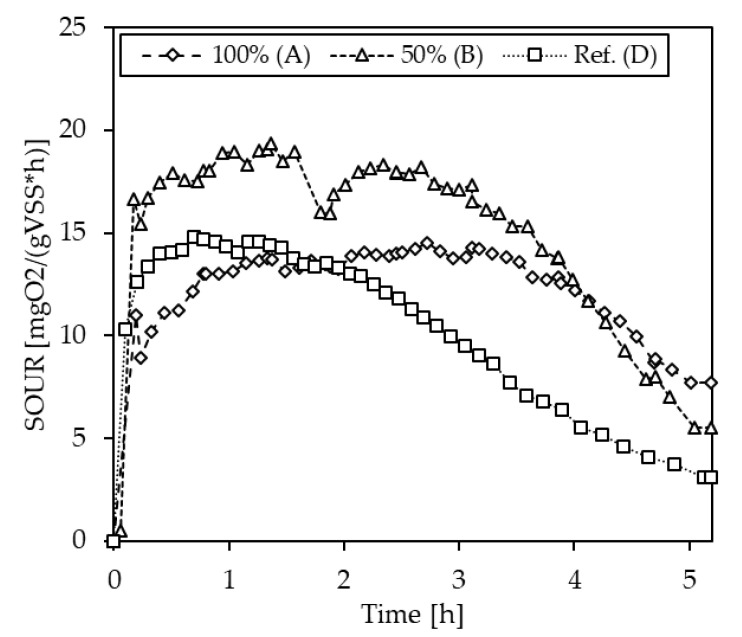
SOUR over the course of the inhibition test with S-1.

**Figure A5:**
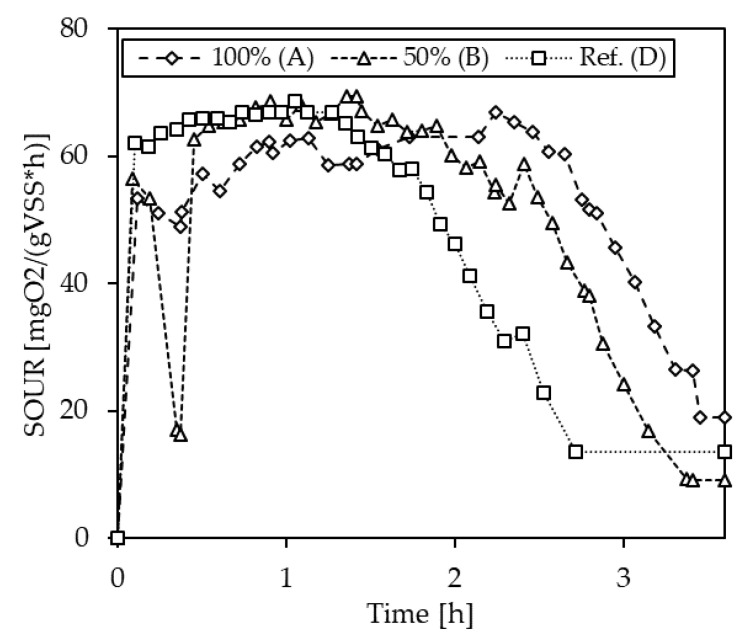
SOUR over the course of the inhibition test with S-2.

**Figure A6:**
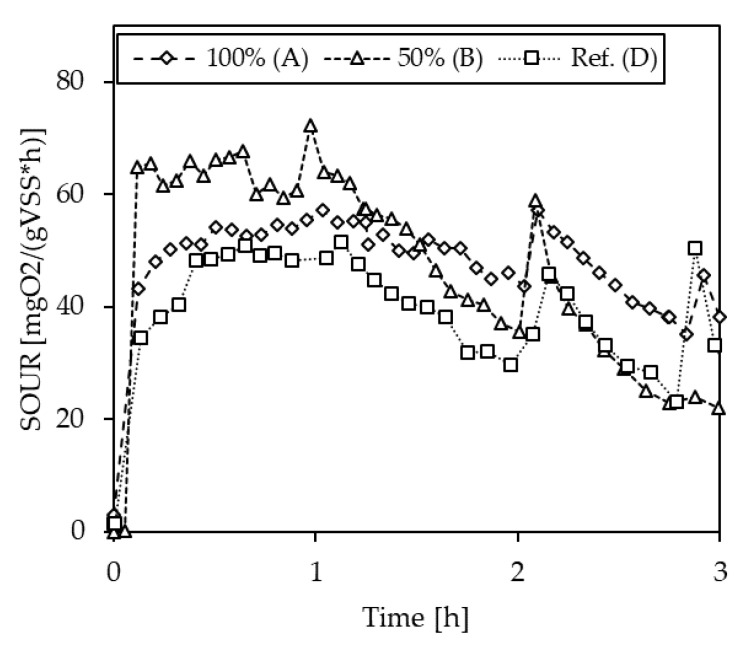
SOUR over the course of the inhibition test with S-4.

Even though a small negative deviation in activity (<5%) is present in most tests with slurry samples, which is a normal phenomenon in microbiological experiments, no inhibition of the nitritation process could be observed. Testing slurry S-1, the maximum SOUR of the reference reaches 14.4 mgO2/gVSS/h. Reactor A (100% sample) reaches a maximum SOUR of 14.4 mgO2/gVSS/h while reactor B (diluted sample 1:2) reaches 18.4 mgO2/gVSS/h. This translates to a deviation to the reference of 2.3% for reactor A. Reactor B even shows a 28.0% higher activity than the reference. In the batch test with slurry S-2, reactor A (100% sample) with a maximum SOUR of 63.4 mgO2/gVSS/h lies 4.2% below the maximum SOUR registered in the reference (66.2 mgO2/gVSS/h)—not yet considered an inhibition. Reactor B (diluted sample 1:2), registering at 66.5 mgO2/gVSS/h, lies on the same level as the reference with a SOUR elevated by 0.4%. Surprisingly, the activity in the reactors supplied with slurry S-4 exceeds the reference in both cases (undiluted and diluted 1:2). Here, the baseline of the reference for maximum SOUR lies at 49.0 mgO2/gVSS/h. Reactor A shows a maximum SOUR of 55.3 mgO2/gVSS/h, which is 12.7% higher than the reference. As in S-1, the diluted sample scores even higher with 66.0 mgO2/gVSS/h, 34.5% higher than the reference. Table 5 gives an overview of the registered maximum SOUR of the inhibition batch tests with pig slurries. The development of SOUR over the course of the experiments is illustrated in Figure A4, Figure A5 and Figure A6 in Appendix A. The registered SOUR and pH of the experiments are listed in the Supplementary Materials in Tables S2–S4.

### 3.2. Characterization and of Examination of Vapor Condensates

#### 3.2.1. Composition of Vapor Condensates

The considered samples show a great range in every parameter. Suspended solids vary widely between the samples, being as low as 0.5 mg/L and as high as 700 mg/L, with shares of organics represented by a LOI of 33.3–86.0%. The samples higher in TSS are the samples with a higher degree in drying, supporting the claim that a high degree of drying results in dustier vapors [28]. The tendency of a higher COD_t_ can be observed in samples with an elevated TSS, with COD_t_ ranging from 70 mg/L up to 8950 mg/L. COD_s_ makes up between 42.7% and 98.3% of COD_t_. With NH_4_-N making up between 68.5% and 94.9% of TN, the range varies vastly from 59 to 2300 mg/L. The COD_s_:NH_4_-N ratio varies between 0.2 and 1.8, not reaching 5.8 in any sample underlining the need for a carbon-independent treatment. TA:NH_4_-N ratio ranges from 0.04 to 0.19 mmol/mg, allowing for a theoretical oxidation of over 56% of the containing NH_4_-N in 4 out of 6 samples. With a pH of up to 10.18, some samples need to be especially considered for FA concentrations if selected for treatment [5].

Table A3 in Appendix A shows a comprehensive overview of all measured values. A detailed comparison of the measurements with the literature is presented under Section 3.2.3.

#### 3.2.2. Condensate Monitoring

The data supplied spans over the years 2018, 2021 and 2022. As in slurry monitoring, the composition varies with almost every parameter expect the pH which is also fairly stable in the condensate. Table 6 shows an overview of the values measured in three years of condensate monitoring with minima, maxima, mean values and (relative) standard deviation.

**Figure 5:**
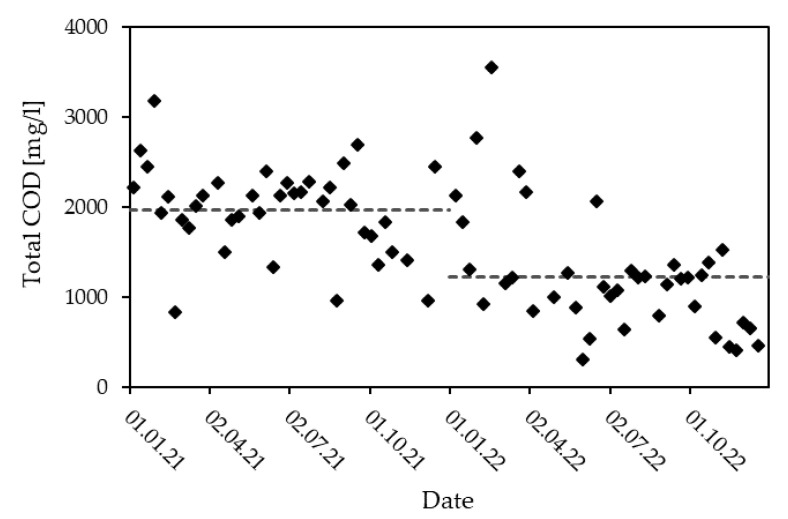
COD_t_ measurements (◊) of condensate monitoring with the annual means of 2021 and 2022 (dotted lines).

The pH, EC, LOI, the Biological Oxygen Demand after 5 days (BOD_5_) and total Kjeldahl nitrogen (TKN) do not differ drastically in terms of their variation and annual mean when comparing the three years considered. While mean TSS is similar in all years, the maximum measured in 2018 lies far below the maxima of 2021 and 2022. Moreover, a rise in COD_t_ from 2018 to 2021 is apparent, as well as a small drop in mean NH_4_-N. Unfortunately, no information on any operational changes in this time period was supplied. Noticeably, there is also a drop in COD_t_ between 2021 and 2022 of 740 mg/L in the annul means, a 36.7% decrease. Figure 5 shows the measured COD_t_ in 2021 and 2022, as well as the annual means represented by a dotted line. The data shows two fairly stable levels with a period of greater variability around the turn of the year. In November 2021, the degree of drying was reduced from 42% to 39% and in the beginning of 2022 the polymer, used to facilitate sludge dewatering, was changed. Moreover, the use of glycerol as a co-substrate was drastically reduced in the beginning of 2022. The decrease in degree of drying is not pronounced enough for a reduction in COD_t,_ as described by Brautlecht [28]. The drastic reduction in glycerol as co-substrate makes for a better explanation, with glycerol having an extremely high COD of up to 1600 g/L [50]. The COD_t_:NH_4_-N ratio in the condensates ranged from 0.4 to 1.7 in 2018, from 0.5 to 1.7 in 2021 and from 0.2 to 1.9 in 2022, with an annual mean of 0.8, 1.0 and 0.6, respectively. As in the samples of the characterization, this favors a carbon-independent treatment. Additionally, the COD_t_ seems not to be readily degradable as the mean BOD_5_: COD_t_ ratios of 0.21 (2018), 0.15 (2021) and 0.23 (2022) suggest.

All values recorded in the condensate monitoring can be found in the Supplementary Materials in Table S5.

#### 3.2.3. Condensate Quality—An Overview of COD_t_, COD_s_ and NH_4_-N

Regarding the current literature, no clear picture on the expectable quality of vapor condensates derived from sewage sludge drying can be drawn. For TA only 6 values could be found. Karwowska, Sperczyńska et al. [30] reported a range from 14.9 to 17.5 mmol/L. Beier, Mahning et al. [27] reported values of 30.5, 35.1, 46.5, 122 and 128.5 mmol/L, which translates to a TA:NH_4_-N ratio of 0.09, 0.07, 0.06, 0.07 and 0.07 mmol/L, respectively, ensuring a theoretical oxidation of over 56% of NH_4_-N in only one sample. The TA values fit well with the findings of this study. TA was found to be between 11.0 and 73.4 mmol/L, and only in this study was TA:NH_4_-N found to be more favorable for deammonification with 4 out of 6 samples reaching the necessary TA:NH_4_-N ratio of 0.0784 mmol/L. 

**Figure 6:**
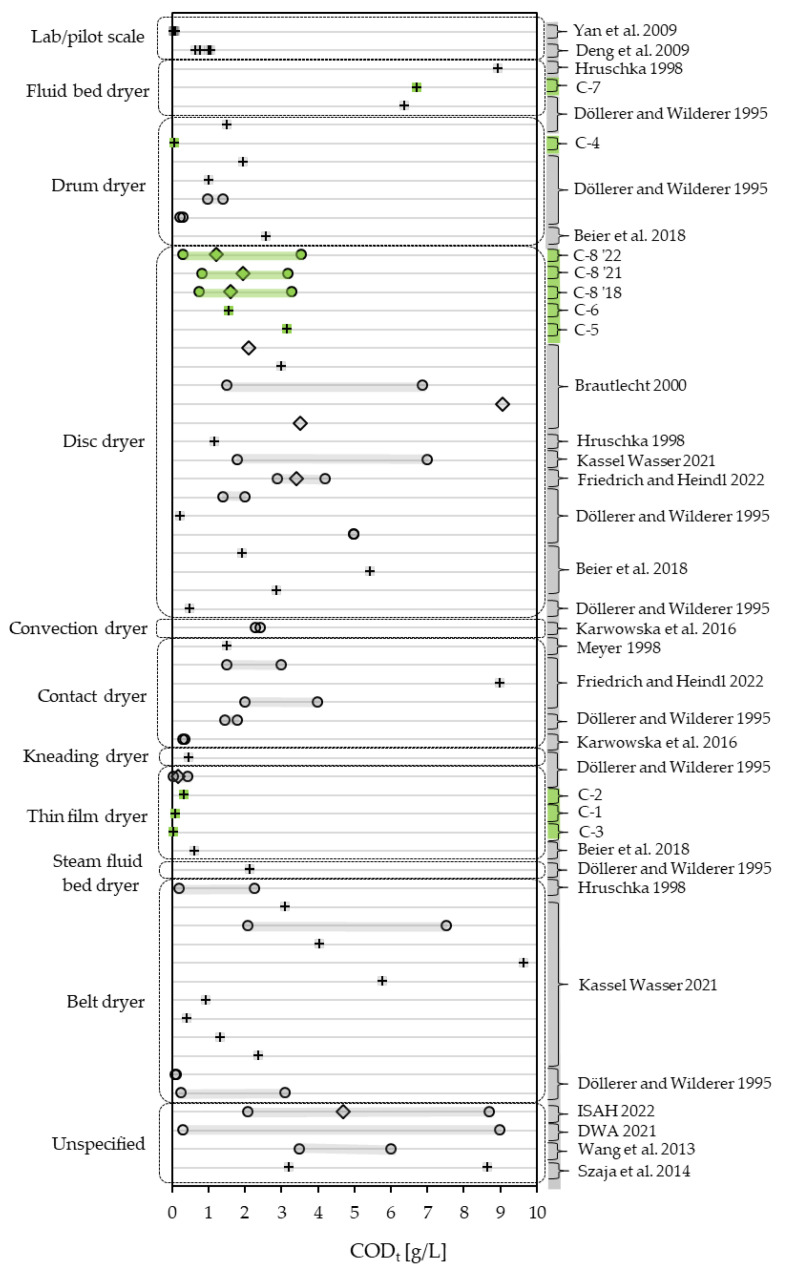
COD_t_ of condensates measured in this study (green) compared to literature values (grey) [4,27,28,29,30,33,51,52,53,54,55,56,57,58], differentiating between one-time samples (+), mean values (◊) and ranges (o).

When comparing the COD_t_ measured in this study with the literature dealing with full-scale dryers, a great variation in COD_t_ becomes apparent. The broadest range reported in the literature spans from 0.3 to 9 g/L and includes almost all values regarded in this study, though ranges from single operations tend to be less broad [29,51,52,53]. The range is stated by the German Association for Water, Wastewater and Waste (DWA) [54] as a generally expectable range, not regarding the type of dryer. In this study, COD_t_ ranges from 0.054 to 8.950 g/L. In the literature, the lowest reported COD_t_ lies at 0.059 g/L, being the low end of a range reported by Döllerer and Wilderer [29] of a continuous operation of a thin-film dryer with a maximum of 0.443 g/L and an annual average of 0.177 g/L. The highest value found in literature, for a large-scale dryer, is a one-time sample of a belt dryer scoring 9.647 g/L [4]. Figure 6 gives a comprehensive overview of the COD_t_ found in the examined samples and the condensate monitoring set in context with the literature, differentiating by dryer type. The widest range of COD_t_ can be found in condensates from belt dryers, spanning almost over the whole range presented in the literature, with 0.107 g/L [29] as its lowest value. The most records for COD_t_ in condensates derive from disc dryers. Mostly the COD_t_ lies below 5 g/L, both one-time samples and the ranges recorded in this study’s condensate monitoring. Where data on a continuous drying operation of disc dryers is available, the COD_t_ spans over a minimum range of 2.334 g/L (C-8 ‘21) and a maximum range of 5.375 g/L [28]. Regarding the ranges found in large-scale drying operations, the effect of temperature on COD_t_ reported from lab experiments by Deng, Yan et al. [55] and Yan, Deng et al. [56] is dwarfed. Data on drum dryers show a maximum of 2.573 g/L COD_t_ [27]. Noticeably, COD_t_ in condensates derived from thin-film dryers score lowest in the comparison with a maximum of 0.628 g/L [27]. On the other end lie the condensates derived from fluid bed dryers, with a minimum in one-time samples of 6.37 g/L [29] and a maximum of 8.95 g/L (C-7).

**Figure 7:**
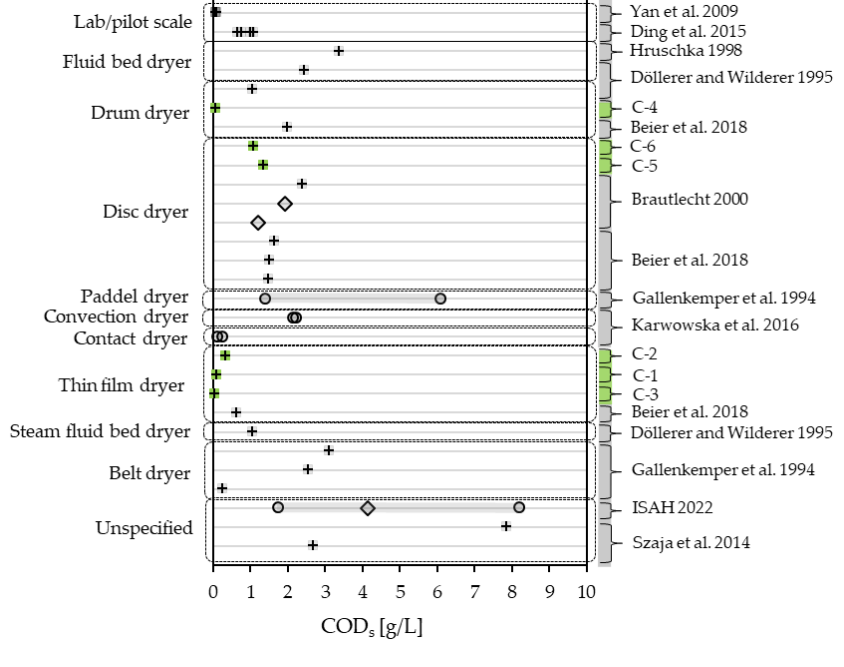
COD_s_ of condensates measured in this study (green) compared to the literature values (grey) [27,28,29,30,51,57,58,59,60], differentiating between one-time samples (+), mean values (◊) and ranges (o).

Even though condensates are less loaded with solids compared to slurries, a lowering of COD_t_ is still achievable with de-dusting or filtration. While most of the condensates where data on COD_t_ and COD_s_ are available have a portion of up 80 to 98% COD_s_ in COD_t_, there are condensates with as little as 21% COD_s_ [28]. COD_s_ in the condensates range less widely than the COD_t_. The lowest value measured in this study is 0.053 g/L (C-3), while the lowest reported in the literature is 0.109 g/L [30] as the low end of a range for contact dryers. The maximum value was the high end of an operating range with 8.215 g/L [57]. Operating ranges show a variation over a span of 4.7 g/L [59] and 6.45 g/L [57]. With COD_s_ in the literature, mostly being reported as one-time samples, it makes for a difficult placement of the measured values in this study. Figure 7 shows the COD_s_ measured in this study and COD_s_ presented in the literature, differentiating by dryer type. The COD_s_ in condensates derived from disc dryers lie between 1.0 and 2.5 g/L with six one-time samples (S-5, S-6, [27,28]) and two mean values [28]. As with COD_t_, the values available for thin-film dryers score the lowest with a maximum of 0.621 g/L [27]. Except for one, all one-time samples considered (6 samples + 15 literature values) lie below 3.5 g/L, the exception being a one-time sample reported by Szaja, Aguilar et al. [58] with a COD_s_ of 7.85 g/L.

**Figure 8:**
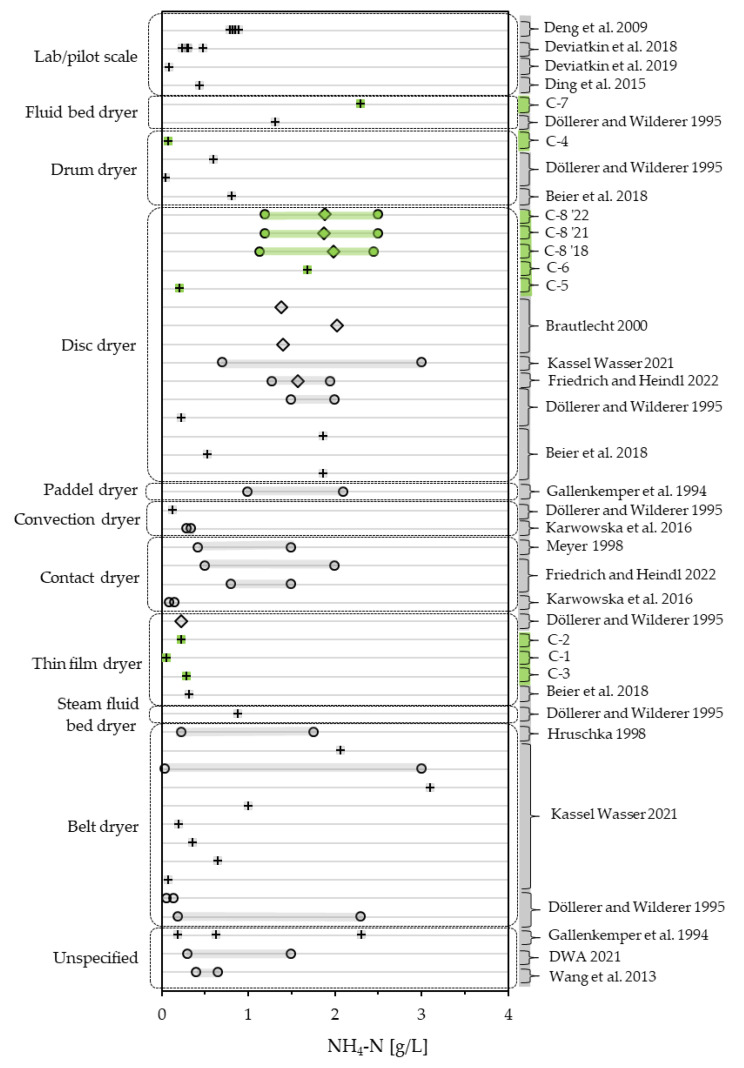
NH_4_-N of condensates measured in this study (green) compared to the literature values (grey) [4,27,28,29,30,33,51,52,53,54,55,58,60,61,62], differentiating between one-time samples (+), mean values (◊) and ranges (o).

As the other parameters do, NH_4_-N values in the literature spread over a wide range from 0.039 g/L to 3.11 g/L [4], including all one-time samples measured in this study as well the monitoring data. Almost half of the values of large-scale dryers considered in this study (21 of 48) exceed the range for the expectable range of NH_4_-N in condensates of 0.3 to 1.5 g/L, stated by DWA [54], these values being one-time samples [4,27,29,59] or the high ends of ranges [4,29,51,52,59] reported in literature as well as C-7 and the annual means in monitoring data from this study. Except for one one-time measurements (3.11 g/L [4]) and the high ends of two ranges with 3 g/L [4], all NH_4_-N values considered lie below 2.5 g/L. This also includes the values reported in lab experiments [55,60,61,62]. Figure 8 shows the values measured and recorded in this study in comparison to the values found in the literature, differentiating by dryer type. The monitoring data fit in well, considering other values found for disc dryers. NH_4_-N minima and maxima in the recorded data span over a range of 1.31 g/L (C-8 ‘18) and 1.30 g/L (C-8 ‘21, C-8 ‘22) and lie in between the ranges of 0.674 g/L [52] and 2.3 g/L [4] reported for disc dryers. Ranges reported in the literature generally span over a minimum of 0.25 g/L [53] up to a maximum span of 2.961 g/L [4]. As with COD_t_ and COD_s_, the range in large-scale operations seem to dwarf the effect of drying temperature on the NH_4_-N content of condensates as reported by Deviatkin, Havukainen [62] and Deng, Yan et al. [55]. Out of the seven one-time samples measured in this study, five values (C-1, C-2, C-3, C-4, C-5) range at the low end of the spectrum of considered values with 0.059, 0.228, 0.286, 0.076 and 0.207 g/L, while C-6 (1.690 g/L) sits fairly in the middle and C-7 (2.3 g/L) ranging on the high. As with COD_t_ and COD_s_, thin-film dryers score noticeably low.

#### 3.2.4. Inhibition Testing with Vapor Condensates

In contrast to inhibition testing with pig slurries, three condensates posed an inhibition to the nitritation process by different degrees. In all inhibitory samples, the effect of inhibition decreases by 54% to 59%, when diluted 1:2. 

Testing condensate C-1 with a low inherent NH_4_-N concentration, reactor A (500 mL of pure sample) only reaches an initial NH_4_-N concentration of 14.5 mg/L, translating to a sludge loading of 0.01 gN/gVSS. The maximum SOUR in reactor A registers at 10.1 mgO2/gVSS/h, 51.2% lower than the reference with 20.7 mgO2/gVSS/h. Reactor B, where the undiluted sample of C-1 was topped with NH_4_Cl the maximum SOUR at 23.0 mgO2/gVSS/h), lies 11.0% higher than the reference. The lack of activity in reactor A seems to be due to a deficient supply of ammonium and not the contents of the condensate itself. 

**Figure A7:**
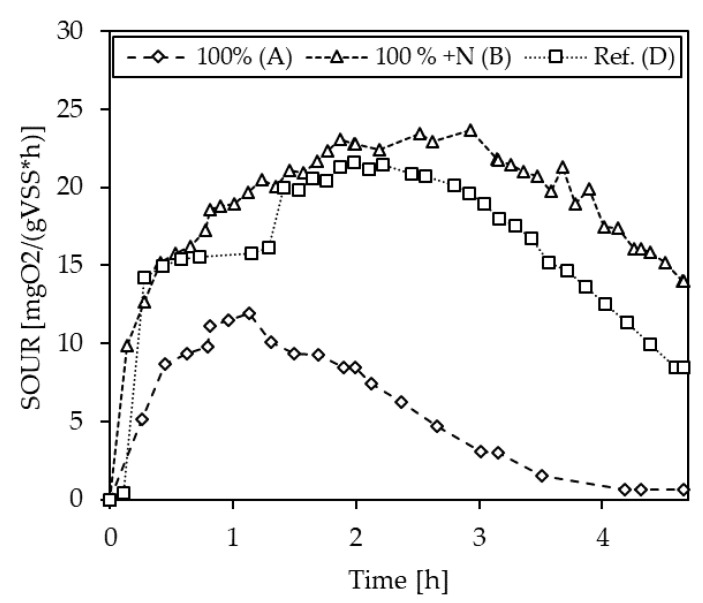
SOUR over the course of the inhibition test with C-1.

**Figure A8:**
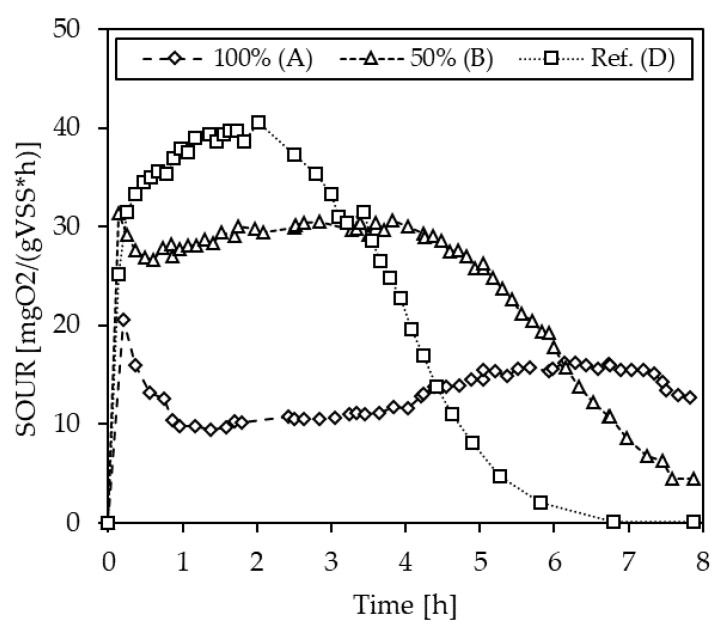
SOUR over the course of the inhibition test with C-2.

**Figure A9:**
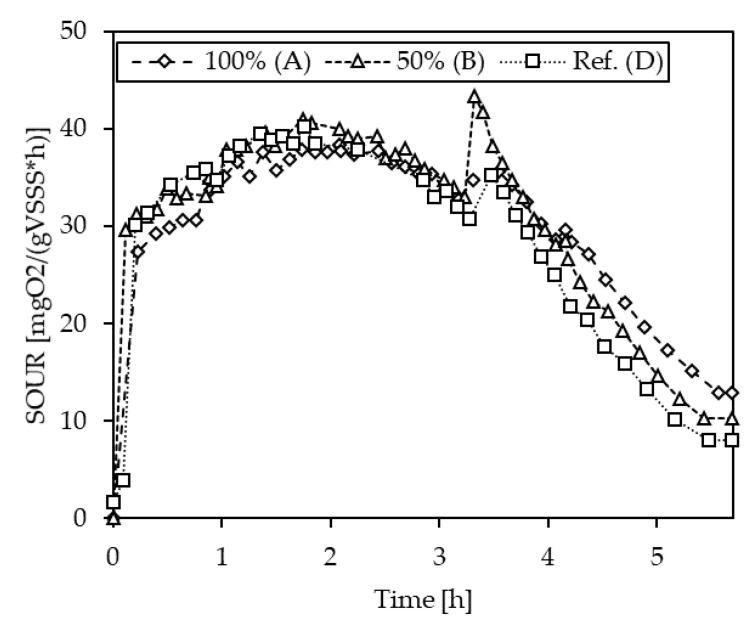
SOUR over the course of the inhibition test with C-3.

**Figure A10:**
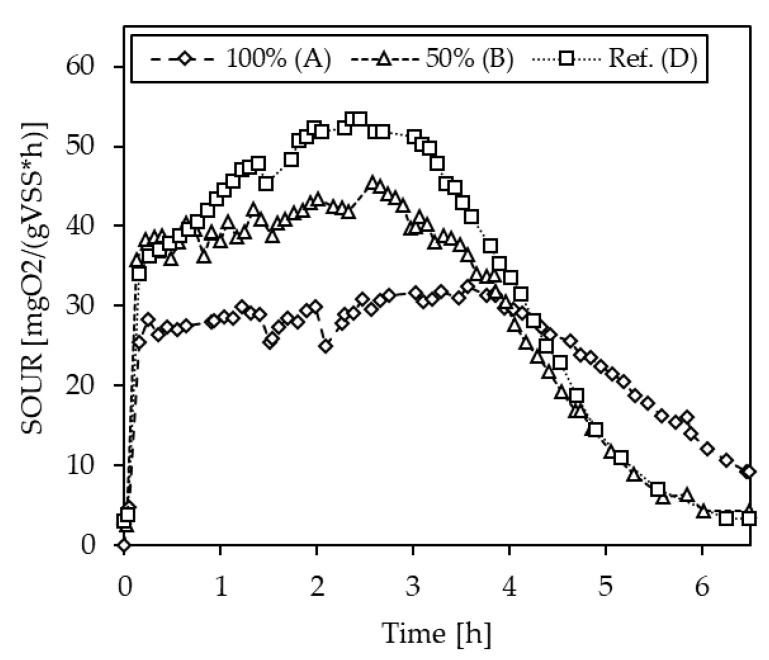
SOUR over the course of the inhibition test with C-6.

In the batch test with condensate C-2, reactor A (100% sample) shows a 60.5% lower maximum SOUR than the reference with 15.6 mgO2/gVSS/h compared to 39.4 mgO2/gVSS/h. In reactor B with the diluted sample (1:2), the maximum SOUR lies at 29.7 mgO2/gVSS/h, 24.5% lower than the reference. This shows a clear inhibition of the nitritation process by C-2. The dilution of C-2 (1:2), however, decreases the inhibitory effect by 59%. In testing condensate C-3, only a slight inhibition is present in reactor A (100% sample) with its maximum SOUR being 5.8% lower than the reference, with 36.5 mgO2/gVSS/h compared to 38.7 mgO2/gVSS/h. Diluting C-3 at a 1:2 ratio results in a decrease of its inhibitory effect as shown by the maximum SOUR in reactor B (38.2 mgO2/gVSS/h) which lies 1.3% under the reference, and is hence not considered to be inhibitory. Condensate C-6 results in an inhibition of the nitritation process by 40.3% with a maximum SOUR of 30.8 mgO2/gVSS/h in reactor A (100% sample), compared to 51.7 mgO2/gVSS/h in the reference. Like in C-2 and C-3, diluting the sample at a 1:2 ratio shows a positive effect on the attainable maximum SOUR, which lies at 42.0 mgO2/gVSS/h in reactor B, 18.7% lower than the reference. This translates to a reduction of the inhibition by 54%. Table 7 gives an overview of the attained maximum SOUR and their deviation to the reference of the condensate batch tests. Condensate C-7 showed no inhibitory effect on the nitritation process as was identified by the company, which supplied the data on their produced condensate. The development of SOUR over the course of the experiments is illustrated in Appendix A in Figure A7, Figure A8, Figure A9 and Figure A10. The registered SOUR and pH of the experiments are listed in the Supplementary Materials in Tables S6–S9. 

## 4. Discussion

The discussion of the obtained results follows the three main objectives of this study: substrate quality, AOB inhibition and deammonification potential.

### 4.1. Discussion of Substrate Quality

The results of this study on the characteristics of pig slurries fit well with the literature and underline the high variability in COD_t_, COD_s_ and NH_4_-N reported. Only the COD_s_:NH_4_-N ratio found in this study, ranging from 1.3 to 4.1, lies clearly lower than the ratios that could be obtained from the literature. Of eight values, only one one-time sample reported by Boursier, Béline et al. [40] showed a ratio of under 3, with the rest ranging from 4.6 over 5.0 up to 12.4 [32,40,45]. This highlights the fact that a COD_s_:NH_4_-N ratio favorable for nitrification/denitrification is not to be taken for granted, even if the current literature suggests otherwise. One influencing factor for the variability in pig slurry quality that is mentioned in the literature is the pigs’ diet. A diet drastically reduced in N can reduce the TN of the pig slurry by up to 0.97 g/L and also reduces NH_3_ emissions [63,64]. Still, the range in NH_4_-N of 1.046 g/L observed in the slurry monitoring (1.87–2.92 g/L) of a continuous piggery operation suggests that peripheral influence factors, such as stabling, storage and use of cleaning water, may have a higher influence in comparing the quality of slurries from different piggery operations [31,32]. In particular, the amount of TS that differs in terms of stabling has a high influence on COD_t_, TP and TN, with the latter potentially being hydrolyzed to NH_4_-N during slurry storage. As the TS, COD_t_ and COD_s_ measurements of pig slurries in this study suggest, a first reduction in COD_t_ can be easily reached via solid–liquid separation. These influencing factors have been investigated for specific piggery operations but not for a categorization of pig slurry qualities, which is helpful in planning possible treatments. From this study, the key figures for pig slurry quality can be derived, though it is not possible to categorize the characteristics in regard to the animal type (piglet, sow, finishing pig) as the peripheral conditions have a higher influence on the quality. 

Considering condensates from sewage sludge drying, an even greater variability in parameters as COD_t_, COD_s_ and NH_4_-N is apparent. While Friedrich and Heindl [52] marked a NH_4_-N concentration of 2 g/L as an extreme example, this study shows that this value is exceeded regularly. In total, 12 of the 52 values (including lab tests) exceed this concentration and additionally 10 values are exceeding 1.5 g/L. The variability of the parameters seems to be greater than often assumed in literature. This variation is not only true when comparing condensates from different drying facilities but also for the long-term operation of one particular dryer. For COD_t_, Brautlecht [28] and Deviatkin, Havukainen et al. [62] reported the influence of drying temperature on COD_t_ which is supported by the reported influence of drying temperature on COD_s_ by Deng, Yan et al. [55] and Yan, Deng et al. [56]. Regarding the data of the long-term condensate monitoring and the data supplied by ISAH [57], this influencing factor seems to be dwarfed by other sludge- and process-related factors, such as the use of co-substrates in sewage sludge digestion. The drying temperature, therefore, is not suitable to categorize condensates and assess substrate quality. Karwowska, Sperczysńska et al. [30] also reported a surprisingly high COD_t_ of up to 2.418 g/L, collected from a medium temperature drier, compared to the condensates from a high temperature drier with a COD_t_ of only up to 0.373 g/L. This supports the claim that other influence factors mask the influence on drying temperature on COD_t_ when comparing different drying operations. For NH_4_-N, the degree of anaerobic stabilization of the sewage sludge has a major influence on NH_4_-N concentration in the condensates, while the temperature does not seem to have any effect [28,65]. This shows that one of the major influences on condensate quality is not the dryer type, degree of drying and drying temperature, but the quality of sewage sludge being dried. Additionally, the type of condenser used can play an important role, with spray condensers diluting the condensate [33]. While this reduces the concentration and any associated difficulties, the load that needs to be treated stays the same. The samples originating from thin-film dryers considered in this study are particularly low in COD_t_, COD_s_ and NH_4_-N. Nevertheless, there is not enough evidence to conclude that the use of thin-film dryers always results in lowly loaded condensates, for no information about the condensers used could be obtained. These numerous influence factors make it virtually impossible to predict condensates quality only based on dryer type, temperature and degree of drying; only tendencies may be concluded. For a better categorization of condensate qualities—needed for better treatment planning—systematic research is direly needed.

### 4.2. Discussion of AOB Inhibition

This study does not show any inhibitions of AOB activity by pig slurry. A direct comparison with the literature is not possible, for no studies systematically examining the influence of raw pig slurry on AOB activity could be found. Even though no inhibition of AOB is to be expected in the treatment of raw pig slurry, the inhibition of AOB reported by Zhang, Lin et al. [22] as a result of the use of anaerobically treated pig manure suggests that the possibility of inhibitory effects of pig slurry might arise in certain cases. Pichel, Moreno et al. [20] also reported inhibitions of anammox bacteria by the direct supply of anaerobically pre-treated pig slurry. With a two-stage set up separating partial nitritation and anammox this could be avoided. In general, inhibition by pig slurry is mainly reported for the anaerobic treatment due to high amounts of free ammonia (FA) [1]. These concentrations do not pose the same problem to the deammonification process as AOB can adapt to high FA-concentrations and the high concentrations also help to suppress the activity of nitrite-oxidizing-bacteria (NOB), ensuring a stable nitritation [17]. High FA-concentrations can also be avoided by process control. This is also true for condensate treatment.

The inhibitions of AOB recorded for condensates in this study fit well with the findings of Beier, Mahnig et al. [27] where, of five samples, three show immediate inhibitory effects and a reduction in the activity of AOB by 30%, 33% and 45%. As in Beier, Mahnig et al. [27], no apparent connection between inhibitory potential and any one parameter considered in this study could be drawn. Karwowska, Sperczyńska et al. [30] suggest a possible inhibitory effect through heavy metals. Unfortunately, this could not be further investigated in this study.

### 4.3. Discussion of Deammonification Potential

While COD_s_:NH_4_-N ratios of up to 12.4 for pig slurries in the literature suggest a good suitability for a treatment via nitrification/denitrification, in this study extremely low values of down to 0.1 were measured, suitable for deammonification. By using COD_s_ as a substitute for COD_b_ in this study, the biodegradability might even be overestimated. The TA:NH_4_-N ratios measured in this study and derived from the literature suggest that an oxidation of approx. 56% an NH_4_-N in the slurries—essential for al substantial nitrogen elimination via anammox—is not of great concern. However, process control measures need to be implemented where an excessive oxidation of ammonium might shift the NH_4_-N:NO_2_-N ratio to be unfavorable for anammox. This could be a halting of the oxidation or, if a two-stage process is used, the supply of ammonium to the anammox via a bypass. From a nutrient perspective pig slurry seems to be well suited for a treatment with deammonification. But in certain cases, the high amount of organics does pose an obstacle for anammox efficiency [16,21]. In addition, the lack of COD_t_ elimination via nitritation and anammox can also hinder certain treatment goals. Therefore, a coupling of the deammonificiation process with some form of COD elimination seems reasonable [6]_._ A first step should be proper solid–liquid separation. Also, currently, the combination of nitritation/anammox with a denitrification is being investigated for anaerobically treated pig slurry/manure with process set-ups and control strategies [24,26]. Due to the high TA present in undigested pig slurry, an increased nitritation (>56% of NH_4_-N) in combination with denitritation might also be of use.

Due to the potentially high NH_4_-N and low COD_s_ content in condensates, they are well suited for deammonification, even though for a full treatment some form of COD removal might be recommended in certain cases [6]. Because no full COD removal is needed for a part stream treatment on a WWTP however, nitritation/anammox seems most adequate. But the low TA:NH_4_-N described in this study implies that for any treatment with a net H^+^ production some form of acid capacity might need to be supplied. Another problem for a biological treatment of condensates poses the inhibition of AOB by certain condensates that could be observed in this study and was also reported by Beier, Mahnig et al. [27]. No clear indicator for inhibitory effects of any condensate could be derived in this study. Further research is direly needed to assume any inhibitory effects of condensates beforehand. Nevertheless, the method for inhibition testing presented in this study allows for a quick and secure assessment of AOB inhibition. In the inhibition experiments, this study found a clear reduction in inhibitory effects by diluting the wastewater sample as also reported by Beier, Mahnig et al. [27]. Apart from any inhibitory substances, the potentially extremely high pH of the condensates can also impair the treatment due to high FA concentrations or by exceeding the pH optimum of the bacteria involved [5]. A combined treatment of condensates with less-contaminated wastewater streams such as process water from sludge dewatering, which could drastically reduce or eliminate any inhibitory effects of condensates as suggested by Brautlecht [28]. For sludge liquor treatment, the deammonification process is already well-established in Germany [66]. The positive effect of mixing the condensates with weaker wastewaters also extends to the high pH reducing the amount of chemicals that might be necessary for pH adjustment. In certain cases, the high pH and NH_4_-N of condensates might be used for NOB suppression in co-treatment. Nevertheless, the production of lowly loaded condensates that do not pose any inhibitions on AOB activity is possible as C-1 shows.

## 5. Conclusions

As shown in this study, pig slurry and vapor condensates show great variability in COD_t_ and NH_4_-N, whereas the quality of pig slurries seems more predictable with its connection to stabling and collection and is set in a narrower range. The COD:NH_4_-N ratio in pig slurry is often unsatisfactory for treatment via nitrification/denitrification. It could be demonstrated that the deammonification process can be beneficially applied for the reduction in nitrogen, especially when COD is removed in a pre-treatment via solid–liquid separation and used to enhance biogas production. A competition between denitrifying bacteria and anammox bacteria for NO_2_-N can be avoided by using a two-stage process where partial nitritation and anammox are separated, especially as no inhibition of AOB was detected with raw pig slurry. In fact, the TA in pig slurries generally supports the deammonification process. Due to the high amount of TA in certain pig slurries, the partial nitritation may exceed the oxidation of 56% of ammonium needed for anammox, shifting the desired NH_4_-N:NO_2_-N ratio of 1:1.32 towards NO_2_-N. The ratio can easily be adjusted by bypassing the nitritation stage with fresh wastewater.

The variability in quality of the condensates exceeds even the variability shown in pig slurries with no direct relation to dryer type or temperature. Concerning the nitrogen removal efficiency, the COD:NH_4_-N ratio found in condensates favors the treatment via deammonification, with no need for an additional COD removal and strong potential for an energy-efficient nitrogen removal process. A problem in condensate treatment is posed by the determined inhibition of up to 60.5% on AOB activity. The method used in this study for inhibition testing gives a quick and reliable result for the necessary classification of the condensate as the inhibitory effects are not easily predictable. In the experiments, with a dilution of 1:2, inhibitory effects could be reduced by more than half; therefore, a combined treatment of condensates and sludge liquor is favorable. 

To summarize, general key figures for pig slurry and condensate quality can be drawn from this study as given above, but for a specific implementation of a deammonifcation process (plant design, control strategy etc.) for pig slurry or condensate treatment it is necessary nevertheless to examine the particular wastewaters. This is especially true for possible inhibitory effects in condensates. In pig slurries and condensates, the up- and downstream processes play a major role in composition and quality, holding potential for quality optimization and assessment. Both effluents are well suited for treatment by deammonification. However, for pig slurry treatment, the process technology needs to be directed towards a solid–liquid separation and/or an integration of COD removal (e.g., up-stream denitrification) in the deammonification process, possible in a two-stage system (as is being studied in the BMBF-funded research project “KompaGG-N” (grant number: 02WQ1516C)). The ammonium concentration of condensates is much lower than in pig slurries but there is a much higher chance of AOB inhibition. Therefore, it is recommended to treat the condensates together with another lower-loaded part stream, which can also compensate the low total alkalinity of condensates and thus strengthen the nitrification.

## Figures and Tables

**Table 1:** Type and origin of the collected pig slurries.

Sample	Animal Type	Feed	Stable	Point of Collection
S-1	Finishing pig	Standard	Slatted floor	Storage tank
S-2	Finishing pig	Standard	Slatted floor	Storage tank
S-3	Finishing pig	Standard	Slatted floor	Storage tank
S-4	Finishing pig	Standard	Slatted floor	Storage tank
S-5	Finishing pig	Standard	Slatted floor	Central channel
S-6	Sow	Standard	Slatted floor	Central channel
S-7	Piglet	Standard	Slatted floor	Central channel
S-8	Finishing pig	Standard	Slatted floor	Central channel
S-9	Finishing pig	Standard	Slatted floor	Storage tank
S-10	Finishing pig	Standard	Slatted floor	Central channel
S-11	Finishing pig	Standard	Slatted floor (animal welfare)	Central channel
S-12	Finishing pig	N-P-reduced	Slatted floor	Storage tank

**Table 2:** Condensate samples and their origin (approximated values).

Sample	TS Sludge	Co-Substrates	Dryer Type	Temperature	Degree of Drying
	[%]			[°C]	[%]
C-1 *	25	Fats (food industry)	Thin film dryer	225–230	50–60
			+ linear dryer	95–100	75–80
C-2 *	25	no	Thin film dryer + disc dryer	190	80–85
C-3	21–32	Yes (unknown)	Thin film dryer	170	42.5
C-4	25	no	Drum dryer	360	93
C-5	25.7	Fats (food industry)	Disc dryer	110–120	93
C-6	20.5	Fats, wet waste, glycerol	Disc dryer	168	39
C-7 **	-	-	Fluid bed dryer	150	98
C-8 ***	20.5	Fats, wet waste, glycerol	Disc dryer	168	39

* Mixed sample of both dryers. ** Data supplied by company. *** 3 years of monitoring data.

**Table 3:** Experimental setting for the inhibition tests.

Reactor	A	B	C	D
VSS [g/L]	1.2–1.3	1.2–1.3	1.2–1.3	1.2–1.3
SL [gN/gVSS]	0.06	0.06	0.06	0.06
DO [mg/L]	2–4	2–4	2–4	2–4
OUR calculation [mg/L]	2.2–3.8	2.2–3.8	2.2–3.8	2.2–3.8
Temperatur [°C]	26	26	26	26
pH	7.5–8	7.5–8	7.5–8	7.5–8
Slurry/condensate	100%	50%	100%	0%
TW with NH_4_Cl	0%	50%	0%	100%
ATU	-	-	86 µmol/L	-

**Table 4:** Minimum, maximum, and mean values measured over a course of 9 months of monitoring pig slurry.

	pH	EC	TS	LOI	COD_t_	COD_s_	NH_4_-N	NO_2_-N	NO_3_-N	TA
	[-]	[mS/cm]	[g/kg]	[%]	[g/L]	[g/L]	[g/L]	[mg/L]	[mg/L]	[mmol/L]
Min.	7.65	16.00	11.3	40.1	6.775	2.750	1.870	0.00	5.41	166
Max.	8.08	29.25	17.7	68.9	29.750	8.610	2.916	2.79	67.00	532
Mean	7.83	22.41	14.7	50.5	13.144	5.373	2.327	0.29	29.91	291
SD	0.10	3.93	2.1	8.8	4.471	1.669	0.290	0.52	13.3	65
%RSD	1.3%	17.6%	14.4%	17.4%	34.0%	31.6%	12.5%	176.7%	44.6%	22.3%
*n*	30	30	7	7	26	24	30	30	30	30

**Table 5:** Maximum SOUR and deviation to the reference identified in AOB inhibition tests with pig slurries.

		Max. SOUR	Deviation to Ref.
		[mgO2/gVSS/h]	[%]
S-1	A (100%)	14.0	−2.3
	B (50%)	18.4	28.0
	D (Ref.)	14.4	-
S-2	A (100%)	63.4	−4.2
	B (50%)	66.5	0.4
	D (Ref.)	66.2	-
S-4	A (100%)	55.3	12.7
	B (50%)	66.0	34.5
	D (Ref.)	49.0	-

**Table 6:** Minimum, maximum and mean values measured for condensate monitoring in 2021 and 2022.

		pH	EC	TSS	LOI	COD_t_	BOD_5_	TKN	NH_4_-N
		[-]	[mS/cm]	[mg/L]	[%]	[mg/L]	[mg/L]	[mg/L]	[mg/L]
2018	Min.	9.0	3.420	0	2.6	747	120	1150	1140
	Max.	9.9	11.800	1250	99.4	3300	680	2510	2450
	Mean	9.5	7.412	94	80.3	1613	317	2077	1992
	SD	0.2	1.880	163	14.8	537	123	328	317
	%RSD	2.0%	26.3%	173.0%	18.4%	33.3%	38.9%	15.8%	15.9%
	*n*	42	41	343	343	41	36	37	35
2021	Min.	9.2	3.360	0	3.4	836	100	1490	1200
	Max.	10.1	10.600	3820	99.3	3180	490	2730	2500
	Mean	9.7	6.443	76	85.2	1967	281	1997	1879
	SD	0.2	1.641	285	13.4	487	93	268	274
	%RSD	1.8%	25.5%	374.7%	15.7%	24.7%	33.1%	13.4%	14.6%
	*n*	38	38	317	321	38	37	38	38
2022	Min.	9.4	3.050	1	8.6	308	74	1290	1200
	Max.	9.9	9.430	3280	100.0	3550	610	2590	2500
	Mean	9.7	6.269	103	86.0	1227	275	2034	1894
	SD	0.1	1.580	327	13.8	668	128	338	324
	%RSD	1.4%	25.2%	316.9%	16.0%	54.4%	46.6%	16.6%	17.1%
	*n*	39	39	312	312	39	32	38	36

**Table 7:** Maximum SOUR and deviation to the reference identified in AOB inhibition tests with condensates.

		Max. SOUR	Deviation to Ref.
		[mgO2/gVSS/h]	[%]
C-1	A (100%)	10.1	−51.2
	B (100% + N)	23.0	11.0
	D (Ref.)	20.7	-
C-2	A (100%)	15.6	−60.5
	B (50%)	29.7	−24.5
	D (Ref.)	39.4	-
C-3	A (100%)	36.5	−5.8
	B (50%)	38.2	−1.3
	D (Ref.)	38.7	-
C-6	A (100%)	30.8	−40.3
	B (50%)	42.0	−18.7
	D (Ref.)	51.7	-
C-7 *	100%	-	0

* No data on SOUR available.

## Data Availability

All relevant data is presented in the Supplementary Materials.

## Supplementary Materials

The following supporting information can be downloaded at: https://www.mdpi.com/2306-5354/10/7/826/s1, Table S1: Measruements of the pig slurry monitoring, Table S2: SOUR and pH recorded in inhibition testing with S-1, Table S3: SOUR and pH recorded in inhibition testing with S-2, Table S4: SOUR and pH recorded in inhibition testing with S-4, Table S5: Measruements of the condensate monitoring (2018, 2021, 2022), Table S6: SOUR and pH recorded in inhibition testing with C-1, Table S7: SOUR and pH recorded in inhibition testing with C-2, Table S8: SOUR and pH recorded in inhibition testing with C-3, Table S9: SOUR and pH recorded in inhibition testing with C-6.

## Appendix A

**Table A1:** List of parameters mentioned in this study with their abbreviations and units.

Abbreviation	Parameter	Unit
COD	Chemical Oxygen Demand	[mg/L; g/L]
COD_t_	Total Chemical Oxygen Demand	[mg/L; g/L]
COD_s_	Soluble Chemical Oxygen Demand	[mg/L; g/L]
COD_b_	Biodegradable Chemical Oxygen Demand	[mg/L; g/L]
BOD_5_	Biological Oxygen Demand after 5 days	[mg/L; g/L]
TOC	Total Organic Carbon	[mg/L; g/L]
TN	Total Nitrogen	[mg/L; g/L]
TKN	Total Kjeldahl Nitrogen	[mg/L]
NH_4_-N	Ammonium nitrogen	[mg/L; g/L]
NO_2_-N	Nitrite nitrogen	[mg/L]
NO_3_-N	Nitrate nitrogen	[mg/L]
FA	Free Ammonia	[mg/L]
TA	Total Alkalinity	[mmol/L]
TP	Total Phosphorous	[mg/L]
PO_4_-P	Orthophosphate phosphorous	[mg/L]
TS	Total Solids	[g/kg]
VS	Volatile Solids	[g/kg]
TSS	Total Suspended Solids	[g/L]
VSS	Volatile Suspended Solids	[g/L]
LOI	Loss On Ignition	[%]
pH	pH value	[-]
EC	Electrical Conductivity	[mS/cm]
DO	Dissolved Oxygen	[mg/L]
OUR	Oxygen Uptake Rate	[mg/L/h]
SOUR	Specific Oxygen Uptake Rate	[mg/gVSS/h]

Table A2 shows the physicochemical characteristics measured in the slurry screening.

**Table A2:** Physicochemical characteristics of the collected pig slurries.

		S-1	S-2	S-3	S-4	S-5	S-6	S-7	S-8	S-9	S-10	S-11	S-12
TS	[g/kg]	15.9	51.8	12.5	-	125.1	5.4	39.5	78.3	46.3	92.2	100.6	42.1
LOI	[%]	69.2	69.5	51.2	-	71.5	61.1	67.1	81.4	72.8	59.1	81.9	65.6
pH	[-]	7.96	7.16	-	-	7.31	7.48	7.67	7.40	8.05	8.07	7.34	7.91
COD_t_	[g/L]	18.51	18.36	11.09	12.84	3.13	3.16	12.80	5.67	47.70	3.94	89.50	41.60
COD_s_	[g/L]	2.53	3.24	6.16	5.72	0.79	1.29	3.67	0.36	0.50	3.48	19.40	8.28
TN	[g/L]	4.17	2.54	3.56	2.93	5.54	0.72	2.05	5.77	5.32	4.75	8.84	4.26
NH_4_-N	[g/L]	2.20	0.82	2.91	2.50	2.35	0.65	1.29	3.26	2.94	1.86	2.92	2.58
NO_2_-N	[mg/L]	0.5	ND	ND	ND	ND	ND	ND	ND	ND	ND	ND	ND
NO_3_-N	[mg/L]	13.2	3.7	23.4	22.9	24.5	16.3	18.8	13.7	9.1	9.1	16.8	35.8
TP	[mg/L]	346	1010	109	124	2720	70	267	1360	1130	3320	1830	1030
PO_4_-P	[mg/L]	50	27	45	-	138	51	112	268	161	175	284	220
TA	[mmol/L]	383	75	198	201	286	70	129	263	256	209	206	259
COD_s_:COD_t_	[%]	13.7	17.7	55.5	44.6	25.3	40.8	28.7	6.3	1.0	88.3	21.7	43.1
COD_s_:NH_4_-N	[-]	1.1	4.0	2.1	2.3	0.3	2.0	2.8	0.1	0.2	1.9	6.6	3.2
NH_4_-:TN	[%]	52.8	32.3	81.6	85.3	42.3	91.1	63.1	56.5	55.3	39.2	33.0	60.6
TA:NH_4_-N	[mmol/mg]	0.17	0.09	0.07	0.08	0.12	0.11	0.10	0.09	0.11	0.07	0.10	0.12

ND: not detectable.

The following Figure A1, Figure A2 and Figure A3 show the variation in COD, NH_4_-N and TA in the period of pig slurry monitoring.

Figure A4, Figure A5 and Figure A6 show the development of the SOUR—corrected for endogenic and heterotrophic respiration—in each experiment. The higher activity of (B) is very prominent in Figure A4, exceeding the SOUR of (A) and (D) which climax on the same level, though at different durations. The sudden drop in SOUR in reactor B in Figure A5 is due to a malfunction of the stirrer and a temporary settling of the sludge. The spikes in SOUR in Figure A6 correspond to a sudden adjustment of the pH of about 0.5 towards alkalinity. The vicinity of the maximum SOUR reached in each reactor is clearly visible. Even though the curve described by the SOUR of the reactors in Figure A6 is not as smooth as in the other batch tests, the SOUR of the reference (D) constantly lies under the SOUR of (A) and (B).

Table A3 gives an overview of the physicochemical characteristics of the vapor condensates.

**Table A3:** Physicochemical characteristics of the considered condensates.

		C-1	C-2	C-3	C-4	C-5	C-6	C-7	C-8^18^ *	C-8^21^ *	C-8^22^ *
TSS	[mg/L]	60	50	0.5	16.9	700	62.7	-	94	76	103
LOI	[%]	47.8	78.2	ND	33.3	68.0	83.0	-	80.2	85.2	86.0
pH	[-]	7.27	9.70	10.18	8.92	8.64	9.57	9.3	9.5	9.7	9.7
EC	[mS/cm]	1.564	0.818	0.714	1.230	1.998	4.310	10.039	7.412	6.443	6.269
COD_t_	[mg/L]	108	348	54	70	3158	1560	8950	1613	1967	1227
COD_s_	[mg/L]	104	324	53	60	1347	1072	-	-	-	-
TN	[mg/L]	66	261	343	91	302	1780	2600	-	-	-
NH_4_-N	[mg/L]	59	228	286	76	207	1690	2300	1992	1879	1894
NO_2_-N	[mg/L]	0.02	0.03	0.36	0.80	0.68	0.32	-	-	-	-
NO_3_-N	[mg/L]	1.02	1.33	2.90	0.34	4.50	0.92	-	-	-	-
TP	[mg/L]	38.40	2.43	0.01	0.45	1.51	1.74	120	1.7	1.1	1.6
PO_4_-P	[mg/L]	38.40	2.40	ND	0.33	1.42	1.41	40	-	-	-
TA	[mmol/L]	11.0	13.5	22.8	11.8	18.5	73.4	-	-	-	-
COD_s_:COD_t_	[%]	96.3	93.1	98.3	86.2	42.7	68.7	-	-	-	-
COD_s_:NH_4_-N	[-]	1.8	1.4	0.2	0.8	6.5	0.6	-	-	-	-
NH_4_-:TN	[%]	89.4	87.4	83.4	83.6	68.5	94.9	88.5	-	-	-
TA:NH_4_-N	[mmol/mg]	0.19	0.06	0.08	0.15	0.09	0.04	-	-	-	-

* annual mean

Figure A7, Figure A8, Figure A9 and Figure A10 show the development of the SOUR—corrected for endogenic and heterotrophic respiration—of the batch tests with the condensates C-1, C-2, C-3 and C-6, respectively. The development of the SOUR (A) in Figure A7 shows clear signs of insufficient nutrient supply by increasing to a lesser level and decreasing quite early due to depletion of available nitrogen. The inhibitory effect is well portrayed in Figure A8 and Figure A10 with a lesser maximum SOUR in (A) and (B) and a prolonged period of activity where nutrient depletion shows up later in time. In Figure A9, the SOUR of (A), (B) and (D) follow the same course with (A) being the lowest curve. As in the slurry tests, the spike between hour 3 and 4 is due to a rather drastic pH adjustment of approx. 0.5.
