# Correction: Reiter, J.; Beier, M. Deammonification Potential of Pig Slurries and Vapor Condensates from Sewage Sludge Drying—Substrate Quality and Inhibition. *Bioengineering* 2023, *10*, 826

**DOI:** 10.3390/bioengineering11050424

**Published:** 2024-04-25

**Authors:** Johannes Reiter, Maike Beier

**Affiliations:** Institute of Sanitary Engineering and Waste Management (ISAH), Faculty of Civil Engineering and Geodetic Science, Leibniz University Hannover, Welfengarten 1, 30167 Hanover, Germany; beier@isah.uni-hannover.de

In the original publication [1], there was a mistake in Table 2. Both typographical errors are in the temperature column of Table 2 and in the line of sample C-1. Instead of 255–230 and 95–10, it should read 225–230 and 95–100. The corrected version of Table 2 appears below:

The authors state that the scientific conclusions are unaffected. This correction was approved by the Academic Editor. The original publication has also been updated.

## Figures and Tables

**Table 2:** Condensate samples and their origin (approximated values).

Sample	TS Sludge	Co- Substrates	Dryer Type	Temperature	Degree of Drying
	[%]			[°C]	[%]
C-1 *	25	Fats (food industry)	Thin film dryer	225–230	50–60
			+ linear dryer	95–100	75–80
C-2 *	25	no	Thin film dryer + disc dryer	190	80–85
C-3	21–32	Yes (unknown)	Thin film dryer	170	42.5
C-4	25	no	Drum dryer	360	93
C-5	25.7	Fats (food industry)	Disc dryer	110–120	93
C-6	20.5	Fats, wet waste, glycerol	Disc dryer	168	39
C-7 **	-	-	Fluid bed dryer	150	98
C-8 ***	20.5	Fats, wet waste, glycerol	Disc dryer	168	39

* Mixed sample of both dryers. ** Data supplied by company. *** 3 years of monitoring data.
