# Supplementary figures and images for: Deammonification Potential of Pig Slurries and Vapor Condensates from Sewage Sludge Drying—Substrate Quality and Inhibition
Source: Bioengineering (Basel). 2023 Jul 11;10(7):826. doi: 10.3390/bioengineering10070826 (PMC10376242; doi:10.3390/bioengineering10070826)

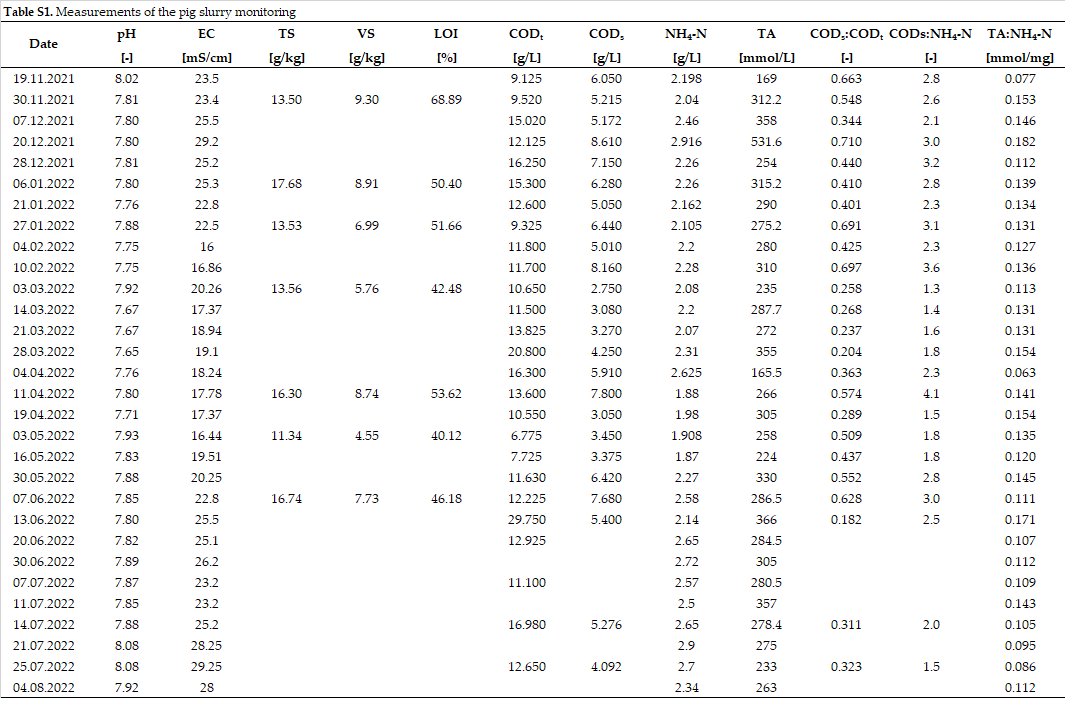

Supplement: Supplementary file 1 [file bioengineering-10-00826-s001.zip › Table_S1.png]

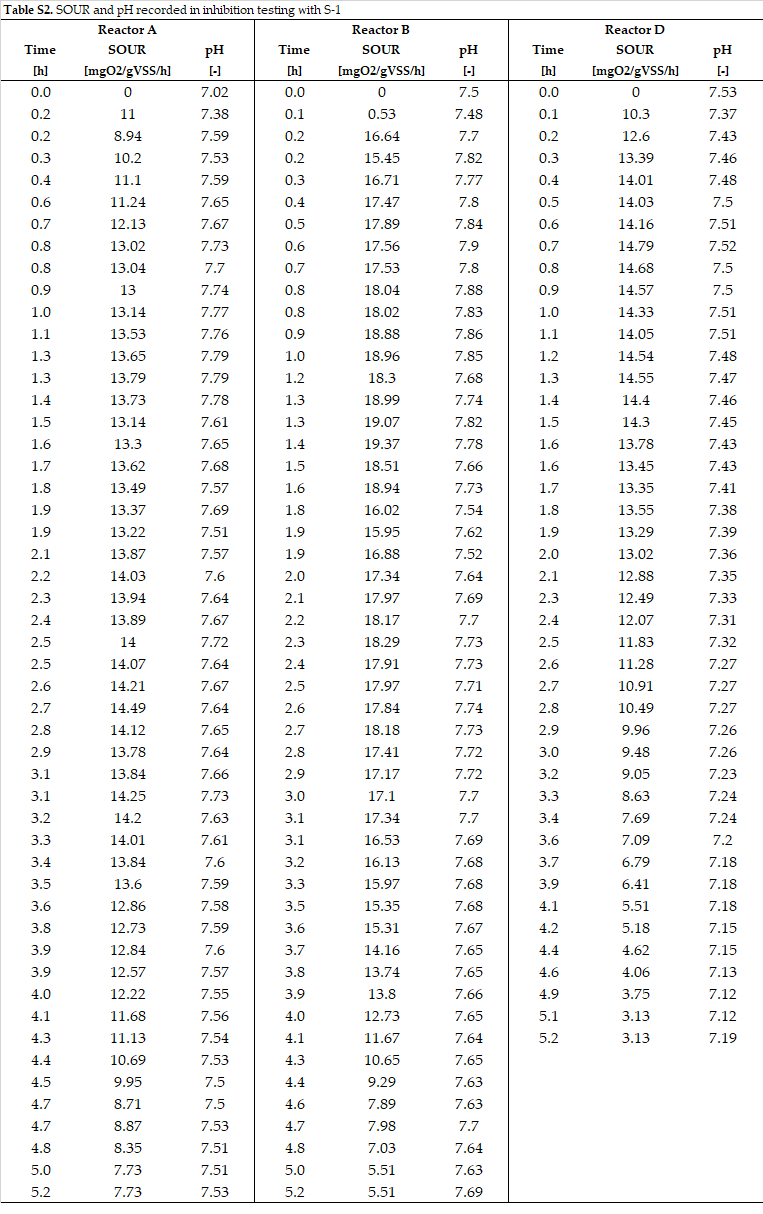

Supplement: Supplementary file 1 [file bioengineering-10-00826-s001.zip › Table_S2.png]

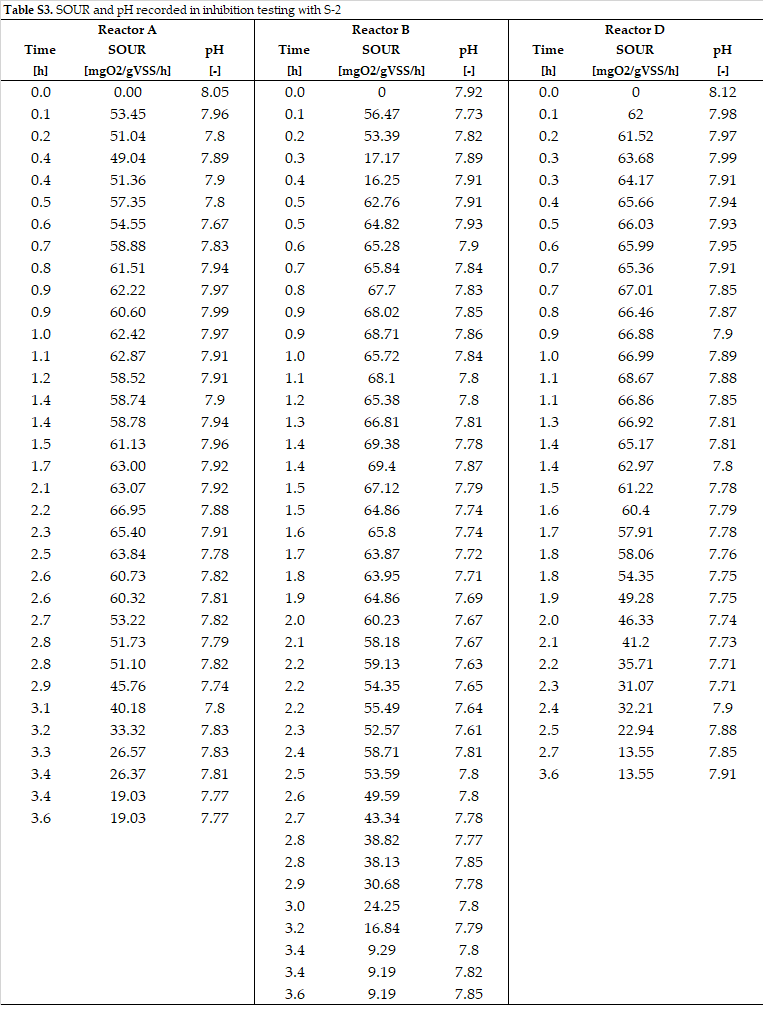

Supplement: Supplementary file 1 [file bioengineering-10-00826-s001.zip › Table_S3.png]

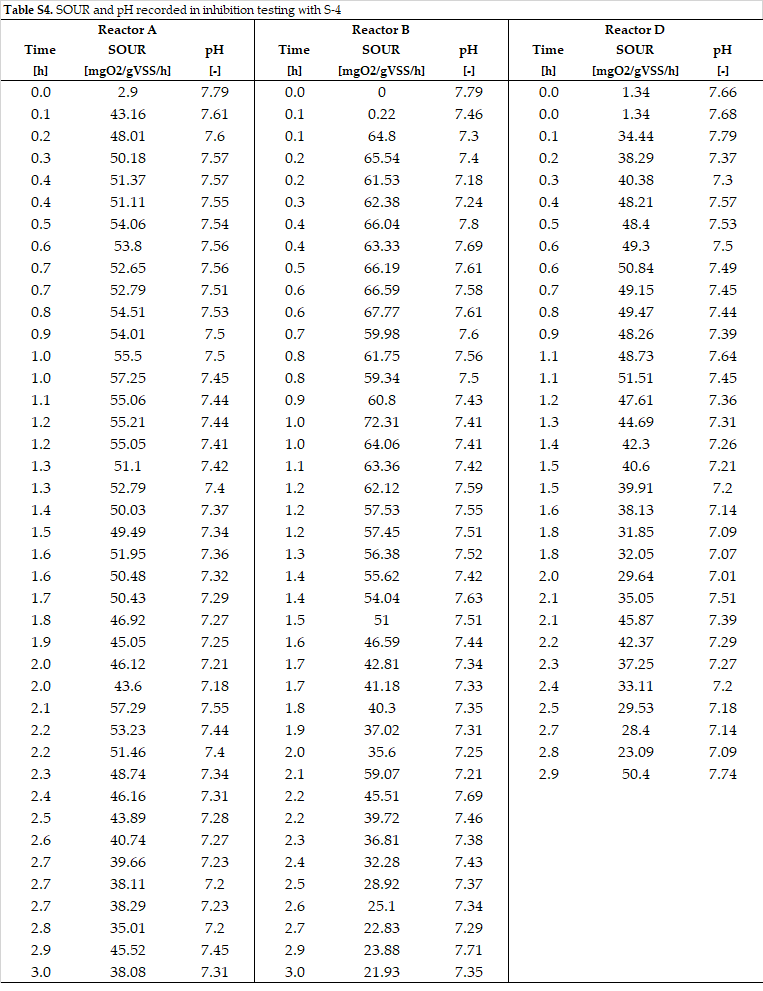

Supplement: Supplementary file 1 [file bioengineering-10-00826-s001.zip › Table_S4.png]

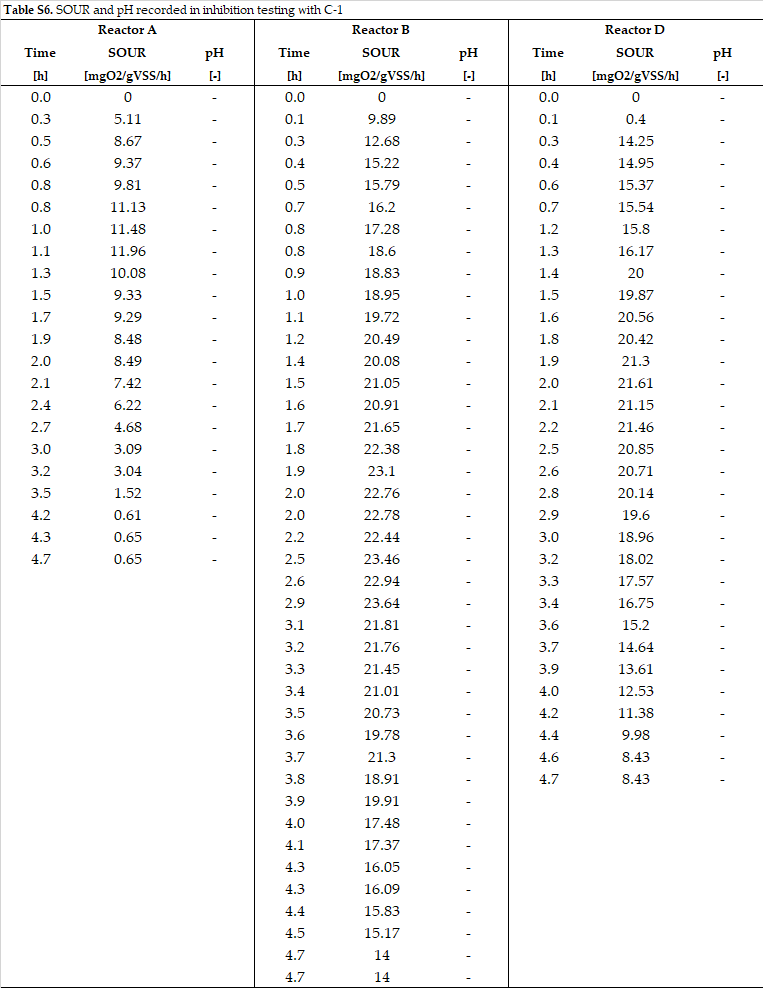

Supplement: Supplementary file 1 [file bioengineering-10-00826-s001.zip › Table_S6.png]

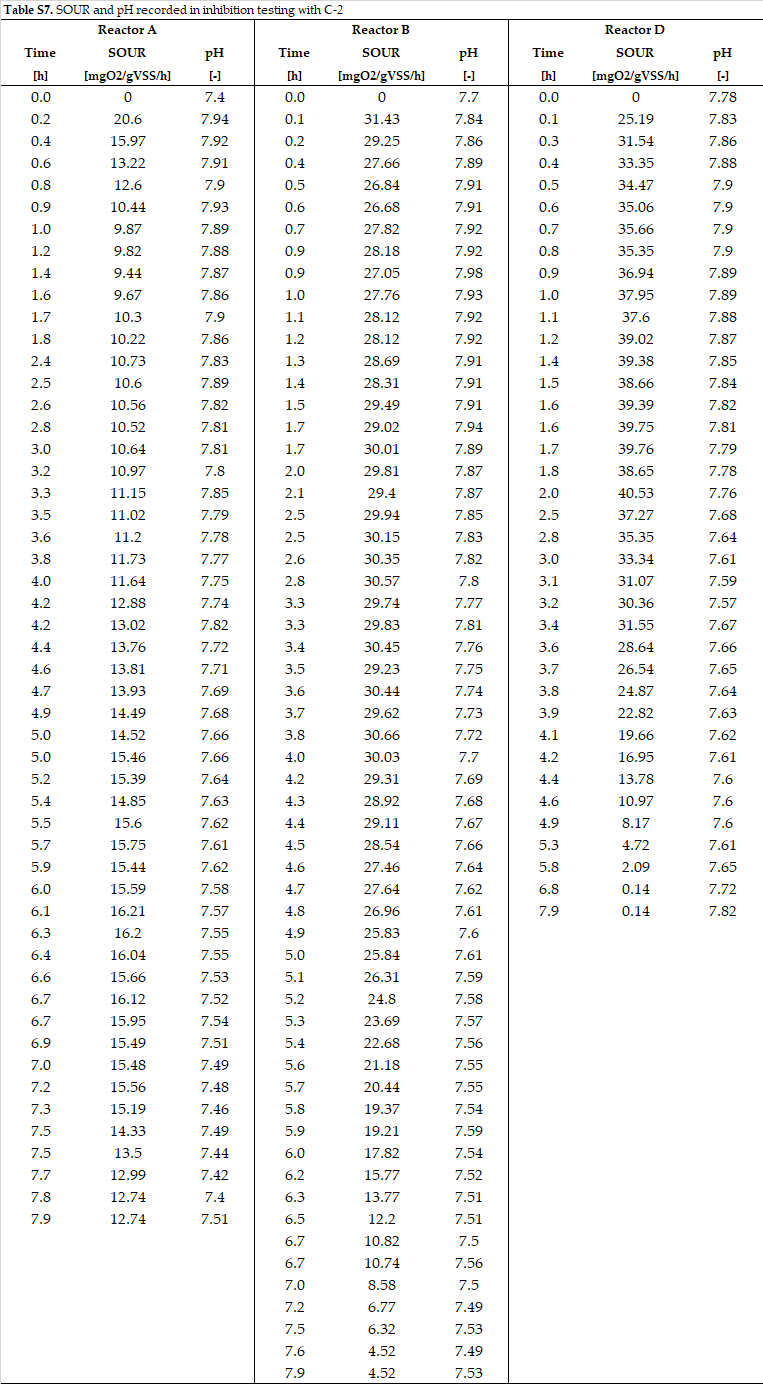

Supplement: Supplementary file 1 [file bioengineering-10-00826-s001.zip › Table_S7.png]

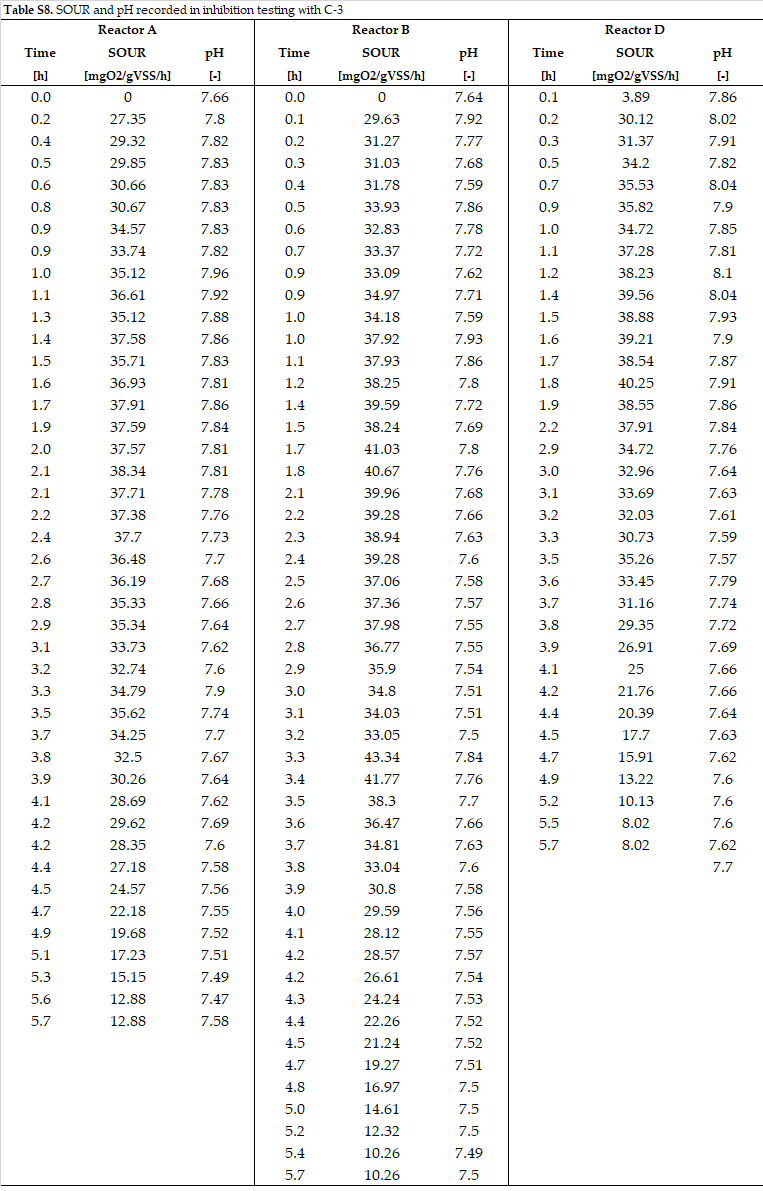

Supplement: Supplementary file 1 [file bioengineering-10-00826-s001.zip › Table_S8.png]

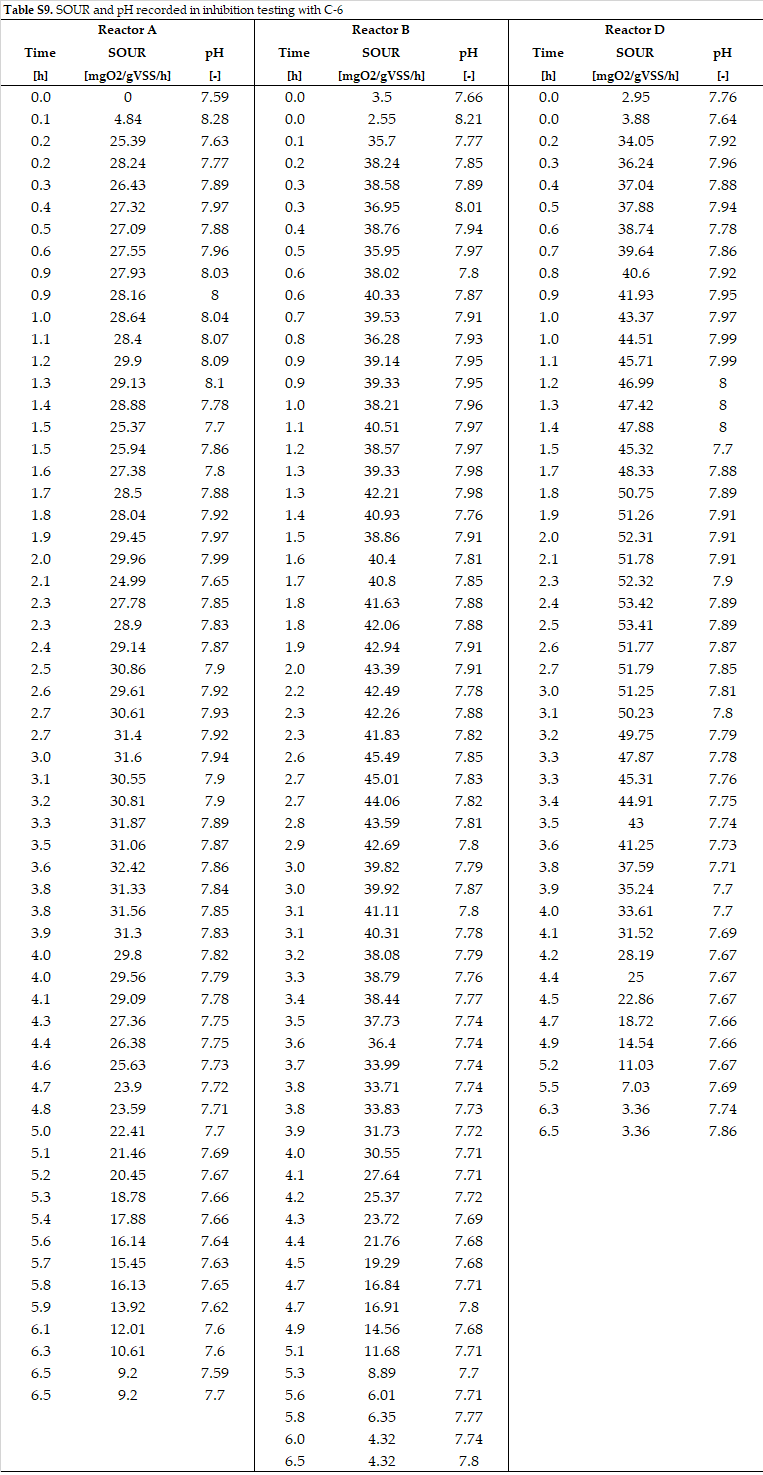

Supplement: Supplementary file 1 [file bioengineering-10-00826-s001.zip › Table_S9.png]
